# Molecular Modeling and In Vitro Functional Analysis of the RGS12 PDZ Domain Variant Associated with High-Penetrance Familial Bipolar Disorder

**DOI:** 10.3390/ijms252111431

**Published:** 2024-10-24

**Authors:** Percy S. Agogo-Mawuli, Joseph Mendez, Emily A. Oestreich, Dustin E. Bosch, David P. Siderovski

**Affiliations:** 1 Department of Pharmacology & Neuroscience, University of North Texas Health Science Center, Fort Worth, TX 76107, USA; percyagogo-mawuli@my.unthsc.edu (P.S.A.-M.);; 2 Department of Biomedical Sciences, Pacific Northwest University of Health Sciences, Yakima, WA 98901, USA; 3 Department of Pathology, Carver College of Medicine, University of Iowa, Iowa City, IA 52242, USA

**Keywords:** alchemical transformation, bipolar disorder, co-immunoprecipitation, docking, free-energy perturbation, genetics, molecular dynamics, surface plasmon resonance

## Abstract

Bipolar disorder’s etiology involves genetics, environmental factors, and gene–environment interactions, underlying its heterogeneous nature and treatment complexity. In 2020, Forstner and colleagues catalogued 378 sequence variants co-segregating with familial bipolar disorder. A notable candidate was an R59Q missense mutation in the PDZ (PSD-95/Dlg1/ZO-1) domain of RGS12. We previously demonstrated that RGS12 loss removes negative regulation on the kappa opioid receptor, disrupting basal ganglia dopamine homeostasis and dampening responses to dopamine-eliciting psychostimulants. Here, we investigated the R59Q variation in the context of potential PDZ domain functional alterations. We first validated a new target for the wildtype RGS12 PDZ domain—the SAPAP3 C-terminus—by molecular docking, surface plasmon resonance (SPR), and co-immunoprecipitation. While initial molecular dynamics (MD) studies predicted negligible effects of the R59Q variation on ligand binding, SPR showed a significant reduction in binding affinity for the three peptide targets tested. AlphaFold2-generated models predicted a modest reduction in protein–peptide interactions, which is consistent with the reduced binding affinity observed by SPR, suggesting that the substituted glutamine side chain may weaken the affinity of RGS12 for its in vivo binding targets, likely through allosteric changes. This difference may adversely affect the CNS signaling related to dynorphin and dopamine in individuals with this R59Q variation, potentially impacting bipolar disorder pathophysiology.

## 1. Introduction

The genetics of bipolar disorder (BD) [1,2,3] are multifaceted, embodying a myriad of factors that contribute to its diverse manifestations and creating formidable obstacles to deciphering its origins and formulating efficacious interventions. This complexity is underscored by a growing body of research indicating that the disorder does not stem from a singular genetic anomaly [4] but rather from a constellation of genetic susceptibilities [5,6,7,8] that interact with environmental stressors and epigenetic modifications. Genome-wide association studies (GWAS), such as those by Mullins et al. [9], have illuminated the polygenic nature of BD, identifying numerous risk alleles [10,11] that, in concert with life events and other external influences, can precipitate the onset of the disorder. Moreover, gene–environment interactions have also been highlighted [12], demonstrating how specific genetic predispositions may render individuals more susceptible to environmental triggers, thereby exacerbating the risk of developing BD. This complexity is further compounded by epigenetic mechanisms, which, according to recent findings (e.g., refs. [13,14]), play a crucial role in mediating the interaction between genetic predispositions and environmental exposures, potentially altering gene expression and impacting disease susceptibility and manifestations, such as suicidal ideation [13]. These current insights collectively underscore the heterogeneous nature of BD, presenting significant challenges to understanding its etiologies and developing targeted, effective treatments.

To identify rare but high-penetrance susceptibility variants for BD, Forstner and colleagues [10] performed whole-exome sequencing of three BD-affected individuals from each of 27 multiply-affected families from Spain and Germany, identifying 378 rare, non-synonymous, and potentially functional variants spanning across 368 different genes and carried by all three BD-affected members in at least one family. An enrichment analysis for all 368 genes revealed significant enrichment for genes also identified in de novo mutation studies of autism and schizophrenia, suggesting a likely genetic overlap with BD for autism and schizophrenia at the rare-sequence-variant level. Within their study [10], two separate gene variations, each causing missense changes (R59Q or Q547H), were found in a gene known to be expressed in select regions of the central nervous system (CNS), namely, the regulator of G protein signaling type 12 (RGS12) [15]. Independently, we previously identified [16] a non-synonymous de novo variant (R702L) within RGS12 by sequencing the exomes of 53 individuals with sporadic schizophrenia (and the exomes of their non-affected parents).

**Figure 1:**
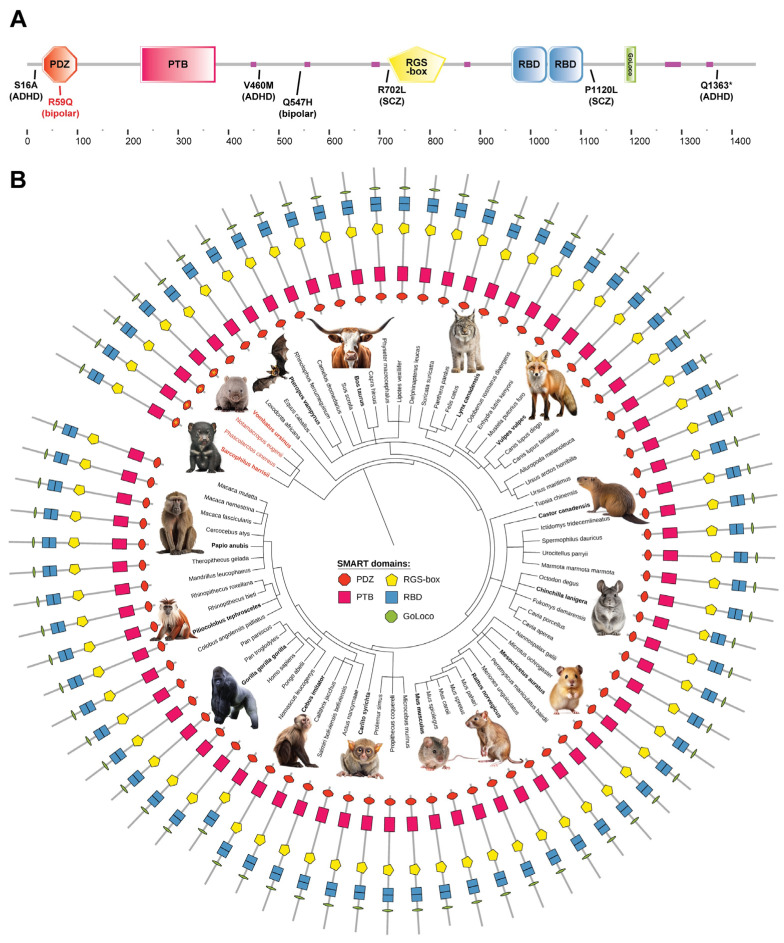
Mental health disorder-associated missense mutations within human RGS12 and its conserved architecture among mammalian species. (**A**) Locations of rare missense mutations associated with attention-deficit/hyperactivity disorder (ADHD), bipolar disorder (BD), or schizophrenia/schizoaffective disorder (SCZ) within the multi-domain architecture of the human RGS12 protein. Multi-domain architecture map of the human RGS12 protein (UniProt O14924, a.k.a. RGS12_HUMAN [1447 aa]) has been placed above an *x*-axis scale of amino acids. Known functional domains within RGS12 include a PDZ (PSD-95/discs-large/ZO-homology) domain, a PTB (phosphotyrosine-binding) domain, an RGS-box (“Regulator of G protein Signaling” domain), a tandem repeat of RBD (Ras-binding) domains, and a GoLoco (Gα_i/o_-locomotion defects) motif; pink regions represent low-complexity polypeptide sequences predicted to lack secondary or higher-order structure. The R59Q missense mutation detected in BD-affected family members [10] is seen to be in the middle of the PDZ domain sequence (highlighted in red). (**B**) Conservation of the multi-domain architecture of RGS12 amongst the orthologues of 74 other mammalian species. Phylogenetic tree of 75 mammalian RGS12 orthologues, represented by respective species’ names and grouped into clades with other mammalian species that share similar evolutionary origins. The protein domain architecture of each RGS12 orthologue is portrayed adjacent to their species nomenclature and consists of the same domain symbols as shown in panel A and derived from the Simple Modular Architecture Research Tool (SMART) database. The RGS12 PDZ domains of four Australian marsupials (names in orange) that contain a lysine rather than an arginine at the position characterized by the human R59Q missense variation are highlighted with yellow asterisks (*) within the orange hexagons that represent PDZ domains.

An earlier schizophrenia-focused trio study [17] reported identifying a de novo P1120L variant of RGS12 within a male proband diagnosed with depressed-subtype schizoaffective disorder. A later genetic study [18] focused on susceptibility to attention-deficit/hyperactivity disorder (ADHD) identified a further three non-synonymous mutations within RGS12 (S16A, V460M, and Q1363* truncation) from the exomic sequencing of Brazilian trios with sporadic ADHD. This set of four independent observations of RGS12 missense mutations associated with mental health disorders [10,16,17,18] (summarized in Figure 1A) strongly supports the hypothesis that genetic variation in the *RGS12* gene could be causal to developing mental health disorders such as BD or, at least, provide new insight into neurotransmitter signaling (dys)function that might direct future BD treatment effectiveness.

RGS12, conserved across mammalian species (Figure 1B), is predominantly expressed in the brains of humans, primates, and rodents (e.g., refs. [19,20]) in multiple regions, including the cortex, claustrum, and caudate putamen—the latter as initially foreshadowed when first cloned in 1997 from a rat striatal cDNA library [15]. Having a central, Gα-binding RGS domain (or “RGS-box”) within its over 1400 amino acids (Figure 1A), RGS12 is the largest member of the RGS family of GTPase-accelerating proteins (GAPs) for heterotrimeric G protein alpha (Gα) subunits [21,22]. We recently established that one of the direct targets for RGS12’s Gα_i/o_ inhibitory action is the striatal kappa opioid receptor (KOR) [23]. RGS12 is most highly expressed within the ventral striatum of the adult mouse brain [24] and serves as a unique marker for its “islands of Calleja” [25]—unique granule cells implicated in the modulation of dopaminergic (DAergic) neurotransmission and thus thought to play crucial roles in reward, motivation, and emotional responses. In concordance with the recent work of Chen et al. [25], our own in situ mRNA hybridization studies suggest that *Rgs12* is co-expressed with *Oprk1* (KOR) transcripts in striatal neurons [23]. Furthermore, endogenous KOR and RGS12 proteins form a complex in mouse ventral striatal explants [23].

The G_i/o_-coupled KOR is highly expressed in the mesolimbic DAergic system and enriched on the presynaptic terminals of the nucleus accumbens within the ventral striatum [26,27,28]. KOR is normally activated during stress by endogenous dynorphins, resulting in analgesia and depressive- and anxiety-like behaviors (i.e., dysphoric/aversive states) [29,30,31,32,33]. KOR activation in the mesolimbic system reduces extracellular DA by (i) inhibiting exocytotic DA release [34,35] and (ii) increasing dopamine transporter (DAT) surface expression and DAT-mediated DA uptake via Gα_i/o_- and mitogen-activated protein kinase (MAPK)-dependent signals [36,37,38]. Our recent fast-scan cyclic voltammetry studies of DA release and reuptake in the nucleus accumbens of RGS12-deficient mice [23] suggest that a loss of RGS12 function disrupts striatal DA release and reuptake kinetics. These disruptions are most likely caused by the enhancement of endogenous KOR signaling upon RGS12 loss, as treatment with the long-lived KOR antagonist norbinaltorphimine reverses this phenotype. Increased DAT expression/function and reduced hyperlocomotion to DAergic-directed psychostimulants exhibited by RGS12-null mice [23,24] are, thus, likely caused by reducing a critical negative influence downstream of KOR activation. This has led to the current hypothesis that reducing RGS12 function in KOR-expressing neurons can mimic G protein-biased KOR agonism, leading to increased DAT function and reduced extracellular dopamine (reviewed in ref. [39]).

Beyond the central, Gα-binding RGS domain that defines RGS12 as a negative regulator of GPCR signaling, several other protein–protein interaction domains within RGS12 have been characterized, at least in vitro, as to their protein-binding specificities (Figure 1A). The C-terminal GoLoco motif is a second Gα interaction site, albeit with a different nucleotide-state selectivity to that of the RGS domain [40,41]. A tandem repeat of Ras-binding domains (RBDs) between the RGS-box and the GoLoco motif (Figure 1A) is involved in nucleotide-dependent interactions with Ras-family GTPases and downstream members of the MAPK family of kinases [42]. An N-terminal phosphotyrosine-binding (PTB) domain is described as belonging to a new domain subclass with unique electrostatics [43]. At the very N-terminus of RGS12 lies a PDZ domain (Figure 1A), named for the first three proteins in which this common structural motif (formerly called a “Discs-large Homologous Region”) was identified, i.e., PSD-95, DlgA, and ZO-1 [44].

The PDZ domain is a protein–protein interaction domain found in a wide range of signal transduction proteins and predominantly found to bind the C-terminal polypeptide “tails” of its interacting partners. A highly conserved G-Φ-G-Φ motif within all PDZ domains (where Φ = a hydrophobic amino-acid) engages the carboxylic acid moiety of PDZ-docking peptide ligands [45,46] (reviewed in ref. [47]). The G-Φ-G-Φ motif is present within the human RGS12 PDZ domain sequence as glycine-31/tyrosine-32/glycine-33/phenylalanine-34 (Supplementary Figure S1; top row, amino acids 30 to 99 of UniProt O14924). Across the PDZ domain sequences of 75 mammalian RGS12 orthologs (Supplementary Figure S1), the arginine residue (“arginine-59”) corresponding to the BD-associated R59Q variation reported by Forstner et al. [10] is also highly conserved, with only 4 of the 75 mammalian species interrogated lacking an arginine at this position, albeit a similar basic amino-acid is present (namely, lysine for four Australian marsupials with unique reproductive and developmental traits, namely *Vombatus ursinus* [common wombat], *Phascolarctos cinereus* [koala bear], *Sarcophilus harrisii* [Tasmanian devil], and *Notamacropus eugenii* [tammar wallaby]).

Having identified a glutamine (Q) substitution at this 59th position within the RGS12 PDZ domain as associated with “Family 085” of their BD proband genetics study, Forstner and colleagues [10] employed five different predictive tools (current to 2020) in an attempt to ascertain a consensus prediction of the functional significance of this R59Q missense variation, namely, SIFT [48], PolyPhen-2 HumDiv and PolyPhen-2 HumVar [49], likelihood ratio testing (LRT; ref. [50]), and MutationTaster [51]. The first two tests predicted that the R59Q variation was “damaging,” the final two conversely predicted a “neutral” change, and PolyPhen-2 HumVar predicted “possibly damaging.” None of these predictions were directly related to the known biological function of the established PDZ domain fold within which the R59Q variation lies.

More modern tools for predicting the consequences of missense mutations are equally focused on overall protein thermodynamic stability (rather than PDZ domain binding function per se) and are equally disparate in their conclusions regarding the R59Q variant. For example, sequence inputs of the first 100 amino acids (spanning the PDZ domain) or full-length human RGS12 (UniProt O14924) into SAAFEC-SEQ [52] returns predicted changes to the overall free energy upon changing arginine-59 to glutamine (ΔΔG of −0.72 and −0.81 kcal/mol, respectively) that were both labeled “destabilizing” (i.e., contrary to the sign of the predicted free-energy change), whereas the more recent PROSTATA tool [53] returns predicted ΔΔG free-energy changes of +0.36 and +0.30 kcal/mol, respectively, suggesting that the R59Q change is destabilizing both to the isolated PDZ domain and to the entire protein. MutPred2, an ensemble of 30 feed-forward neural networks each trained on a set of 53,180 pathogenic and 206,946 unlabeled (putatively neutral) protein variants [54], predicted a less than 50% chance that the variant is pathogenic (MutPred2 score of 0.417) when provided the full-length RGS12 sequence and its R59Q variation.

Using an NMR-derived structural model of the unliganded RGS12 PDZ domain (specifically, conformer “pose 16” from a collection of 20 low-energy conformers within Protein Data Bank record 2KV8; Supplementary Figure S2), the structure-based tools CUPSAT [55] and mCSM [56] provide conflicting predictions. CUPSAT considers the R59Q substitution to be stabilizing to the overall fold, but with the glutamine replacement producing unfavorable torsion angles and a positive change in free energy (ΔΔG of +0.32 kcal/mol), whereas mCSM predicts destabilization in light of a modest overall predicted free-energy change of −0.034 kcal/mol. Again, none of these structure-based predictive tools are specifically attuned to the biological function known for PDZ domains, namely, the binding to short peptide sequences of target protein partners, often at their C-termini, which typically conform to specific tetrapeptide recognition motifs [45,46,47,57].

In our initial report identifying the PDZ domain within RGS12, we characterized its binding selectivity as being directed toward C-terminal (A/S)-T-*X*-(L/V) peptide motifs [19]. Via yeast two-hybrid screens, far-western blotting, and surface plasmon resonance (SPR) biosensor assays [19], specific binding was observed between RGS12 PDZ domain protein fusions and the final pentamer of threonine-serine-threonine-threonine-leucine (TSTTL-COOH) derived from the C-terminal tail of the GPCR interleukin-8 receptor B (CXCR2). Our subsequent report [42], characterizing RGS12 as a neuronal Ras/MAPK signaling scaffold, also suggested that the RGS12 PDZ domain can bind the C-termini of two additional neuronal proteins, namely MEK2 (a middle-tier kinase within the canonical MAPK cascade; ref. [58]) and SAPAP3 (a.k.a. DLGAP3, a synaptic scaffolding protein highly expressed in striatum; ref. [59]). We verified the MEK2 interaction with RGS12 via follow-up SPR and cellular co-immunoprecipitation experiments [42], but the SAPAP3 interaction was not verified at that time beyond describing the initial yeast two-hybrid screening result.

Here, to assess possible functional consequences of the BD-associated R59Q variation within the RGS12 PDZ domain, we first validated the SAPAP3 interaction and then performed in silico molecular dynamics and in vitro SPR binding assays to characterize whether the R59Q variation affects the binding of the RGS12 PDZ domain to one or more of these three protein C-tail targets.

## 2. Results

### 2.1. Establishing a Model for the Liganded Wildtype RGS12 PDZ Domain and the Disposition of Its Arginine-59 Side Chain

Structural models of the RGS12 PDZ domain (PDB id 2KV8), as derived from nuclear magnetic resonance (NMR) imaging of the recombinant protein in solution, have been deposited into the Research Collaboratory for Structural Bioinformatics’ Protein Data Bank (RCSB.org [60]). However, the 20 different low-energy conformers represented within the PDB record 2KV8 (Supplementary Figure S2) each lack a bound C-terminal peptide ligand.

**Figure 2:**
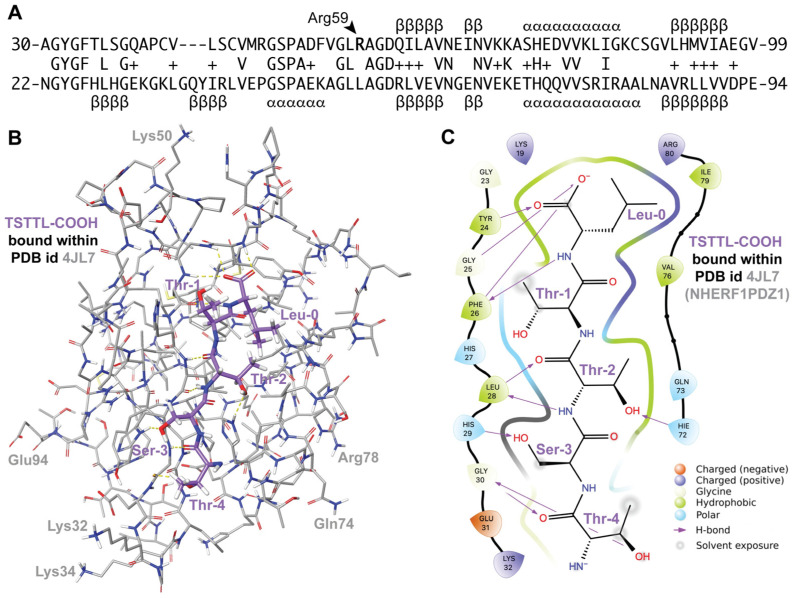
(**A**) Sequence and secondary structure similarity between human RGS12 PDZ domain (top row; aa 30–99 of UniProt O14924) and human NHERF1 PDZ1 domain (bottom row; aa 22–94 of UniProt O14745): −, gap; +, similar amino acid; α, alpha-helical character; β, beta-strand character. Secondary structure derived from PDB IDs 2KV8 and 4JL7, respectively. (**B**,**C**) 3D and 2D representations of the NHERF1 PDZ1 domain/CXCR2 C-tail interaction (“TSTTL-COOH”), rendered via Maestro using PDB id 4JL7. Hydrogen bonds between the PDZ domain and bound pentameric ligand are depicted as yellow dotted lines in panel (**B**) and purple arrows in panel (**C**).

**Figure 3:**
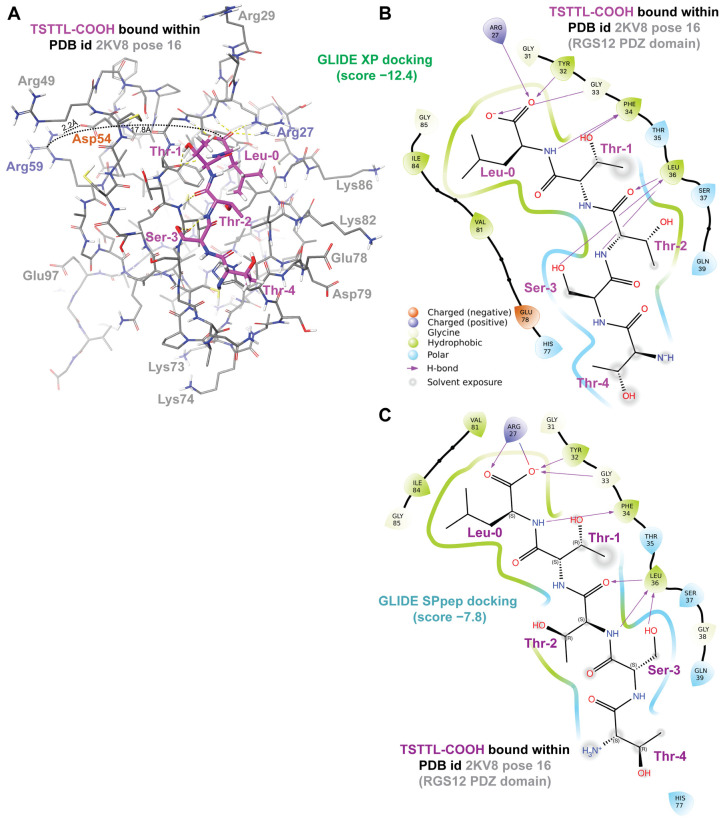
Structural models of the RGS12 PDZ domain/CXCR2 C-tail complex, as derived from GLIDE XP (panels **A**,**B**) and GLIDE SPpep (panel **C**) docking algorithms. (**A**,**B**) Three- and two-dimensional representations, respectively, of the predicted RGS12 PDZ domain/CXCR2 C-tail complex, rendered via Schrödinger’s Maestro after GLIDE XP docking of the free TSTTL-COOH pentameric peptide into the structural coordinates of low-energy pose 16 of PDB id 2KV8. Dotted black lines in panel A represent the distances measured in the 3D model between a hydrogen of the arginine-59 side chain and an oxygen of a potential hydrogen-bonded neighbor (asparagine-54; 2.2 Å) and between the center of the Arg59 side chain’s guanidinium group and the central carbon of the TSTTL-COOH ligand’s carboxylic acid moiety (17.8 Å). (**C**) Two-dimensional representation of the predicted RGS12 PDZ domain/CXCR2 C-tail complex, rendered after GLIDE SPpep docking of the free TSTTL-COOH pentameric peptide into the structural coordinates of low-energy pose 16 of PDB id 2KV8.

To facilitate the computation of likely high-affinity peptide ligands for the RGS12 PDZ domain, and thereby assess the likelihood that the SAPAP3 C-terminal tail is also a high-affinity ligand, we first identified the putative peptide-binding site within these existing “apo” (ligand-free) RGS12 PDZ domain structural models within PDB record 2KV8. As first reported by Snow et al. [19], the polypeptide sequence of the human RGS12 PDZ domain bears homology (Figure 2A) to the first PDZ domain of the human sodium–hydrogen exchange regulatory protein NHERF1 (a.k.a. EBP50 or SLC9A3 regulator 1 [61]). High-resolution crystal structures have been reported [62,63] for the NHERF1 protein’s first PDZ domain bound to the C-terminal tail of the CXCR2 GPCR (i.e., the polypeptide pentamer TSTTL-COOH). We, therefore, used one of these structural models (PBD id 4JL7; Figure 2B,C) to define the presumptive binding site for C-tail peptides within each of the 20 low-energy models of the apo-PDZ domain of RGS12 provided within PDB id 2KV8. Given that the NHERF1 first PDZ domain’s binding surface is generally circumscribed by the residues Tyr24 through His29, His72, Val76, Ile79, and Arg80 (ref. [63] and Figure 2C), a “receptor grid” representing the presumed ligand-binding site of the RGS12 PDZ domain was specified for each of the 20 low-energy conformers (namely, the corresponding RGS12 residues of Tyr32 through Ser37, His77, Val81, Ile84, and Gly85). Using this inferred receptor grid and Schrödinger’s GLIDE SPpep and GLIDE XP docking algorithms [64], in silico docking of the pentamer TSTTL-COOH was then performed for each of the 20 structural models within PDB record 2KV8. Among the best-scoring receptor–ligand pairings (averaging a GLIDE SPpep score of −6.5 and a GLIDE XP score of −8.5; Table 1), low-energy conformer “pose 16” of the RGS12 PDZ domain provided the highest scoring “receptor grid” for the docking of the TSTTL-COOH ligand (GLIDE SPpep score of −7.8 vs GLIDE XP score of −12.4; Figure 3A,B and Table 1).

**Figure 4:**
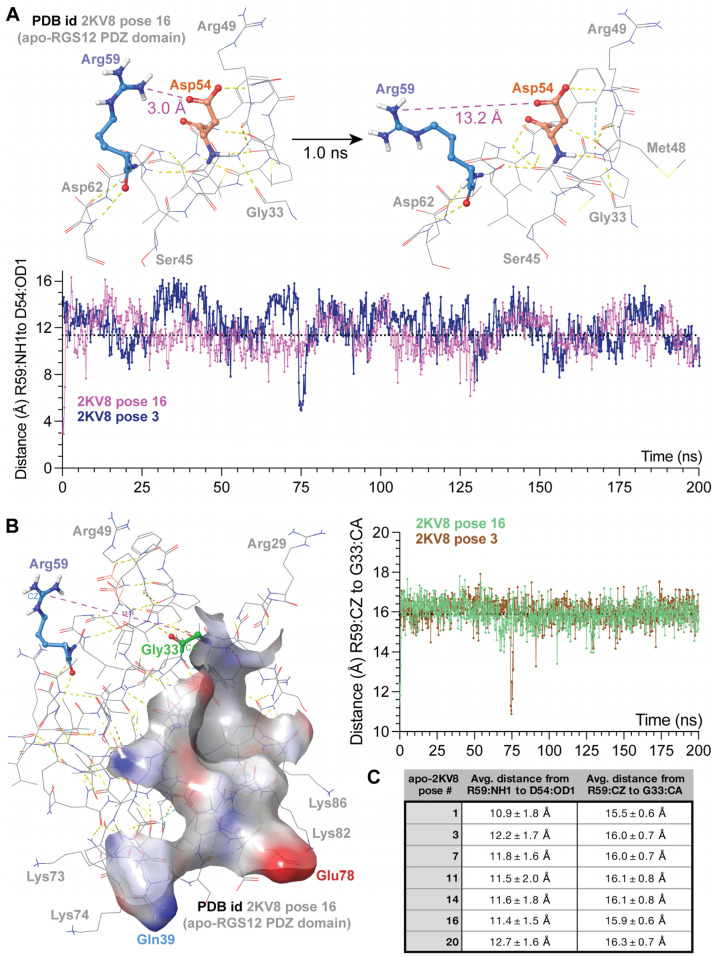
Tracking predicted distances of Arg59 from neighboring Asp54 and the G-Φ-G-Φ motif by MD simulations. (**A**) Depictions of the solvent-accessible arginine-59 (light blue) and asparagine-54 (orange) side chains at the start (time “zero”) of an MD simulation of the unliganded RGS12 PDZ domain (pose 16, PDB id 2KV8) and after 1.0 nanoseconds have elapsed. Plotted underneath is the predicted distance (over 200 ns of MD simulation) between the nearest nitrogen of the arginine-59’s guanidium side chain and the carbonyl–oxygen of the neighboring asparagine-54 side chain within pose 16 (magenta line) and separately for pose 3 (dark blue line). Dotted line indicates average distance over 200 ns of 11.4 ± 1.5 Å (mean ± s.d) for pose 16. (**B**) Depiction of the solvent-accessible arginine-59 and glycine-33 (green; the second glycine of the G-Φ-G-Φ motif) at the start (time “zero”) of an MD simulation of the unliganded RGS12 PDZ domain (empty ligand-binding cavity highlighted in an electrostatics-shaded surface: blue = electropositive and red = electronegative). Plotted to the right is the predicted distance (over 200 ns) between the central carbon (CZ) of the arginine-59’s guanidinium side chain and the alpha-carbon (CA) of glycine-33 for pose 16 (green line) and pose 3 (brown line); dotted line indicates average distance over 200 ns of 15.9 ± 0.6 Å (mean ± s.d) for pose 16. (**C**) Average distances over 200 ns MD trajectories obtained for both residue pairs in separate simulations of other poses of the RGS12 PDZ domain from PDB id 2KV8.

The primacy of the G-Φ-G-Φ motif [62,63] in engaging both the carboxylate and the amide part of the peptide bond at the end of the bound pentamer (e.g., residues Gly23 through Phe26 in NHERF1 PDZ1; Figure 2C) was maintained in predicted docking by GLIDE SPpep and XP algorithms of TSTTL-COOH within the wildtype RGS12 PDZ domain (i.e., residues Gly31 through Phe34; Figure 3B,C). The arginine-59 residue of the wildtype RGS12 PDZ domain, which is changed to glutamine in the BD-associated R59Q variant, is seen to be a considerable distance away from the presumptive TSTTL-COOH binding site in the wildtype RGS12 PDZ domain (>17 Å; Figure 3A). Uniquely for pose 16 of PDB id 2KV8, this arginine-59 side chain is predicted to be hydrogen bonded to a neighboring surface side chain (i.e., Asp54: ~2.2 Å distance between side-chain oxygen acceptor and predicted hydrogen of Arg59’s nitrogen donor; Figure 3A). However, tracking the predicted distance between the oxygen acceptor of Asp54’s side chain and Arg59’s potential nitrogen donor across 200 ns of simulated molecular dynamics suggests that the distance between the possible donor and acceptor averages 11.4 ± 1.5 Å (mean ± s.d.; Figure 4A,C) is a distance incompatible with a persistent hydrogen bond [66]. Using the same 200 ns molecular dynamics trajectory for this pose, the distance between the CZ carbon of Arg59’s side chain is predicted to be an average of 15.9 ± 0.6 Å away from the CA carbon of glycine-33 (in the center of the presumptive G-Φ-G-Φ motif; Figure 4B,C), again suggesting that Arg59 is distant from, and thus not capable of directly influencing, the C-tail binding site within the wildtype RGS12 PDZ domain.

Independent 200 ns MD trajectories for other poses from PDB id 2KV8 confirmed that Arg59 is predicted to be generally distant from both Asp54 and Gly33 (Figure 4C) across all trajectories obtained. Only one pose (pose 3, graphs of Figure 4A,B) demonstrated a very short-lived juxtaposition of Arg59 near Asp54 (~5 Å, Figure 4A) and Gly33 (~11 Å, Figure 4B) at the 75 ns mark in the 200 ns long trajectory. Based on these trajectories, we conclude that the Arg59 side chain (the site of the R59Q variation) is unlikely to interact persistently with other surface side chains, nor directly with residues of the C-tail peptide docking region, especially given that, in the latter, any changes to the function of the peptide docking region upon substitution of the Arg59 residue would likely involve allosteric changes.

### 2.2. In Silico Evaluation of a Third Binding Target for the Wildtype RGS12 PDZ Domain—SAPAP3

**Figure 5:**
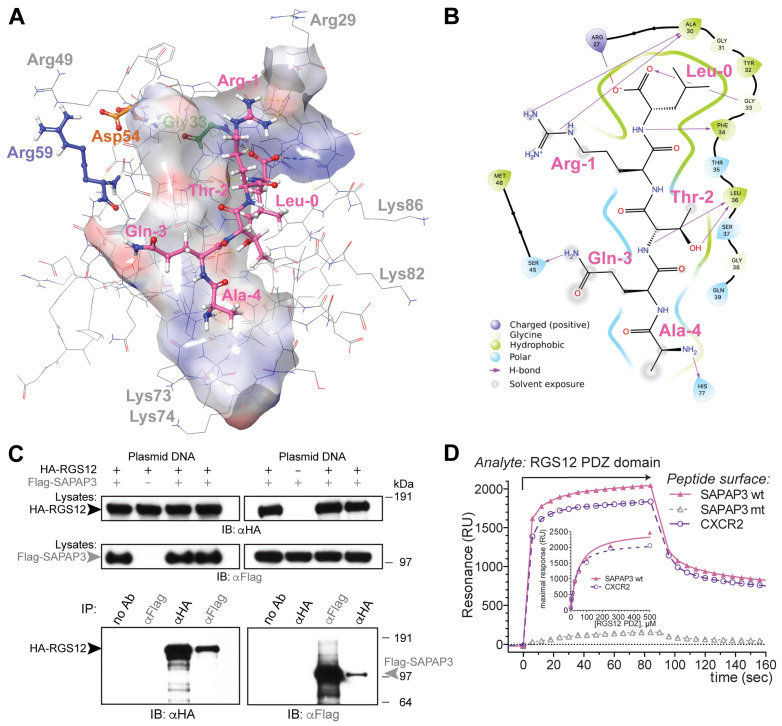
Model of SAPAP3 bound to the RGS12 PDZ domain and in vitro evidence of interaction. (**A**) Three-dimensional electrostatics-shaded surface representation (blue = electropositive, red = electronegative) of the 16th low-energy pose of RGS12 PDZ domain (PDB id 2KV8), as bound by the AQTRL-COOH pentamer that represents the C-terminal tail of the SAPAP3 protein (hydrogen bonds indicated by dashed yellow lines). (**B**) Two-dimensional map, derived from panel A, of hydrogen bond interactions (purple arrows) between the AQTRL-COOH pentamer and RGS12 PDZ domain residues, as predicted by in silico docking (GLIDE XP score −12.1). (**C**) Reciprocal co-immuno-precipitation of HA-epitope tagged RGS12 and Flag-epitope tagged SAPAP3 exogenously expressed in HEK293T cells, confirming previously published observations of an RGS12/SAPAP3 interaction from a yeast two-hybrid screen with N-terminal RGS12 PDZ and PTB domains as the bait molecule and a mouse whole brain cDNA library as the prey [42]. IB, immuno-blotting; IP, immuno-precipitation; no Ab, no primary antibody control; αHA, anti-HA epitope primary antibody; αFLAG, anti-Flag epitope primary antibody; Lysates, whole-cell lysates separated by SDS-PAGE to control for expression and gel-loading; kDa, kiloDaltons (positions of co-electrophoresed marker proteins). (**D**) The PDZ domain of RGS12 binds directly to the C-terminal tail of SAPAP3 in vitro. Surface plasmon resonance (SPR) was performed using separate biosensor surfaces of immobilized, biotinylated peptides from the C-terminal tails of wildtype (wt) SAPAP3 (AQTRL-COOH), a point mutant and capped version of the SAPAP3 C-tail (T964S plus amidated carboxy-terminus; “mt”), or CXCR2 C-tail (TSTTL-COOH) as a positive control for binding [19]. Recombinant RGS12 PDZ domain protein (300 μM; ref. [65]) was used as the analyte. Inset: increasing concentration of RGS12 PDZ domain protein was used as the analyte to determine maximal binding to the wildtype SAPAP3 and CXCR2 C-tail surfaces.

For each of the 20 models within PDB record 2KV8, the presumed peptide-binding site of the RGS12 PDZ domain was patterned from the NHERF1 PDZ1 complex PDB id 4JL7 and then used for further in silico docking of pentamers TRTAV-COOH (the MEK2 C-tail established as a valid target [42]) and AQTRL-COOH (the SAPAP3 C-tail suspected as a target based on yeast two-hybrid results [42]). The low-energy conformer “pose 16” of the RGS12 PDZ domain again provided the highest scoring “receptor grid” for the GLIDE XP docking of both MEK2- and SAPAP3-derived peptides (GLIDE XP scores of −10.8 and −12.1, respectively; Table 1 and Figure 5A,B). Independent docking runs of the same 20 protein poses from PDB id 2KV8 with the same pentameric peptides were also performed using the GLIDE SP-peptide algorithm, a version of the GLIDE standard precision docking protocol with improved parameterization for flexible polypeptides as ligands [67]. As shown in the heat map within Table 1, pose 16 of the RGS12 PDZ domain again provided the highest scoring “receptor grid” for engaging the MEK2-, and SAPAP3-derived peptides (GLIDE SPpep scores of −7.6 and −8.8, respectively; Table 1).

Other protein/peptide docking algorithms were also tested in this study, but none performed as adequately in light of the particular constraints known for the PDZ domain–C-terminal peptide interaction. For example, high cluster-value models of the NHERF1 PDZ domain with the CXCR2 C-tail, obtained from the ClusPro 2.0 webserver (https://cluspro.bu.edu/peptide/index.php; accessed on 24 September 2024), routinely predicted the PDZ domain’s G-Φ-G-Φ motif interacting with closely spaced interpeptide carbonyl and hydroxyl groups rather than the C-terminal carboxylic acid per se (e.g., Figure S3A). ClusPro 2.0 is at an inherent disadvantage for modeling PDZ domain binding by C-tail peptides because it treats the peptide sequence input as within a “polypeptide continuum” (i.e., stripping away the hydroxyl group at the C-tail). Conversely, the best model of the NHERF1 PDZ/CXCR2 C-tail interaction obtained via pepATTRACT (https://bioserv.rpbs.univ-paris-diderot.fr/services/pepATTRACT/; accessed on 24 September 2024) posed the C-tail carboxylic acid too close to the G-Φ-G-Φ motif, leading to multiple steric clashes (Figure S3B).

An independent, free-energy perturbation (FEP) molecular dynamics simulation [68] was performed with pose 16, earlier identified by GLIDE (Table 1) as optimal for peptide docking, to assess the relative binding of the wildtype RGS12 PDZ domain to the three pentameric targets of TSTTL-COOH, TRTAV-COOH, and AQTRL-COOH. These three peptides were related to each other via a network of ‘alchemical’ single amino-acid transformations between the three polypeptide sequences (i.e., a 9-node/18-edge permutation map illustrated in Supplementary Figure S4A). Comparing the best GLIDE docking scores obtained for each pentamer with the relative energy calculations made by the Schrödinger FEP+ MD algorithm (as fully outlined in Figure S4C) suggests that all three methods concur in ranking the MEK2 pentameric C-tail third in likely affinity (Table 2). While the GLIDE XP docking scores and the FEP+ free energy calculations show similar numerical values for each peptide, it is important to note that the GLIDE docking scores, although indexed similarly to Gibb’s free energy, incorporate algorithm-specific penalties and adjustments that prevent direct comparison to true thermodynamic free-energy values. Nevertheless, both the docking and FEP+ predictive methods agree in ranking the MEK2 pentamer (TRTAV-COOH) as having the lowest relative binding affinity to the RGS12 PDZ domain, reinforcing the robustness of this conclusion across multiple computational approaches.

### 2.3. In Vitro Evaluation of SAPAP3 as a Third Binding Target for the Wildtype RGS12 PDZ Domain

To test whether the predicted SAPAP3 C-tail/RGS12 PDZ domain interaction can be detected in vitro, we overexpressed epitope-tagged RGS12 and SAPAP3 proteins by transient cellular co-transfection. We subjected the resultant whole-cell lysates to co-immunoprecipitation tests. The HA-epitope tagged RGS12 full-length protein was seen to co-immuno-precipitate with Flag-epitope tagged SAPAP3 full-length protein when the latter was immunoprecipitated out of whole-cell lysate using an anti-Flag primary antibody (Figure 5C). Also observed was the reciprocal co-immunoprecipitation of SAPAP3 upon immunoprecipitating the HA-RGS12 protein with an anti-HA primary antibody (Figure 5C).

As co-immunoprecipitation indicates complex formation, but not necessarily a direct protein–protein interaction, we then turned to surface plasmon resonance (SPR) to interrogate whether the purified recombinant RGS12 PDZ domain protein demonstrates any direct binding affinity for the isolated C-tail of SAPAP3. We had previously used SPR [65] to establish the direct and selective binding of the isolated rat RGS12 PDZ domain (as a recombinant GST-fusion protein) to the C-terminal tail of rat CXCR2 (i.e., the 12-mer VGSSSAN**TSTTL**-COOH [19]) and the C-terminal tail of rat MEK2 (i.e., the 16-mer RTLRLKQPSTP**TRTAV**-COOH; see ref. [42] in its Supplementary Information section). Using a similar SPR methodology [65], we found here a recombinant RGS12 PDZ domain protein bound specifically to an immobilized 20-mer peptide (SATESADSIEIYIPE**AQTRL**-COOH) representing the C-terminal end of human SAPAP3 protein (Figure 5D and its inset). Using the same SPR assay, no appreciable binding was measured to a point mutant version of this peptide (Thr977-to-serine) that had been additionally amide-capped at its C-terminus (i.e., SATESADSIEIYIPE**AQSRL**-CONH2).

### 2.4. MD Simulations of Wildtype and Variant RGS12 PDZ Models for Ligand Selectivity Changes

**Figure 6:**
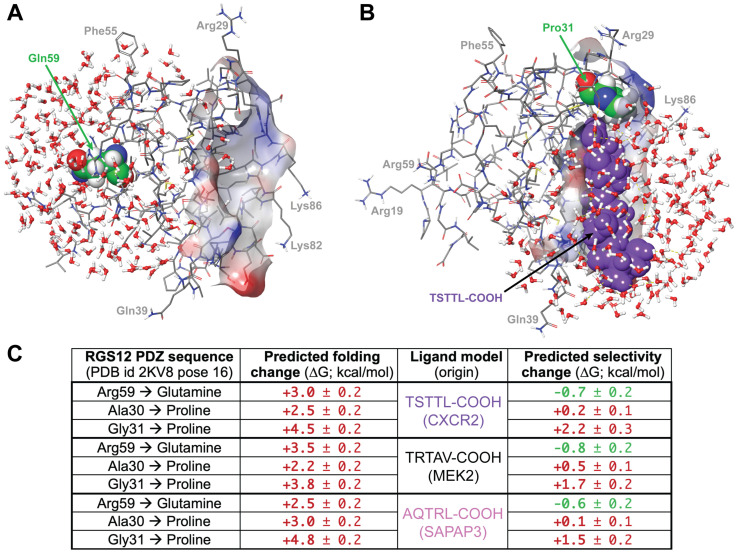
Representative solvation states and results of MD simulations of liganded RGS12 PDZ domain point mutant variants. (**A**) Three-dimensional electrostatic surface representation (blue = electropositive, red = electronegative) of the empty ligand-binding cavity of the 16th low-energy pose of RGS12 PDZ domain (PDB id 2KV8) with an arginine-59-to-glutamine substitution; solvation with 0.15 M NaCl is illustrated in CPK coloring about the glutamine-59 position (Gln59 highlighted in green spheres). (**B**) Three-dimensional electrostatic surface representation (blue = electropositive, red = electronegative) of the ligand-binding cavity of the 16th low-energy pose of RGS12 PDZ domain (PDB id 2KV8) bound to the TSTTL-COOH pentamer (purple spheres) and containing a glycine-31-to-proline substitution (Pro31 highlighted in green spheres); solvation with 0.15 M NaCl is illustrated in CPK coloring about the bound pentameric ligand. (**C**) MD results of predicted free-energy changes (in kcal/mol; values are mean ± std. dev.) to domain folding and to indicated ligand binding using Schrodinger’s FEP Protein Mutation for Ligand Selectivity with default settings. Predictions of destabilization colored in red, and predictions of stabilization colored in green.

To probe whether the BD-associated R59Q variation could affect the ligand-binding selectivity of the RGS12 PDZ domain, for any or all of these three established C-tail peptides, physics-based FEP molecular dynamics simulations were run for single residue mutations within the RGS12 PDZ domain model (e.g., Figure 3A,B and Figure 6A,B), using an FEP+ workflow that employs the Desmond molecular dynamics engine [69,70]. Wildtype RGS12 PDZ domain models, derived from PDB id 2KV8 pose 16 and bound to one of the three docked pentamers (TSTTL-COOH of CXCR2, TRTAV-COOH of MEK2, or AQTRL-COOH of SAPAP3), were first solvated with 200 waters in the region around the single amino acid mutation (e.g., Figure 6A) or peptide-binding site (e.g., Figure 6B). Using Bennett’s approach [71] for estimating the free-energy difference between two canonical ensembles, the change of arginine-59 to glutamine within the RGS12 PDZ domain (“Gln59”; Figure 6A) was predicted to modestly destabilize the folded domain structure (i.e., ΔΔG of between 2.5 to 3.5 kcal/mol; Figure 6C), yet slightly improve the ligand-binding affinity for all three pentamers (i.e., ΔΔG of between −0.6 to −0.8 kcal/mol; Figure 6C).

To calibrate these predictions, a disruptive mutation was separately made to the first glycine of the highly conserved G-Φ-G-Φ motif that engages the carboxylate of peptide ligands [45,46,47], namely, a glycine-31-to-proline mutation (“Pro31”; Figure 6B). This disruptive mutation (G31P) was predicted by the FEP molecular dynamics simulations to lead to a greater level of domain structure destabilization (i.e., ΔΔG of between 3.8 to 4.8 kcal/mol; Figure 6C) and to reduced ligand binding for all three ligands (i.e., ΔΔG of between 1.5 to 2.2 kcal/mol; Figure 6C). A similar change to proline at the neighboring alanine-30 residue before the G-Φ-G-Φ motif was not predicted to have the same level of free-energy changes as for the Gly31-to-proline disruption (Figure 6C). In combination, these particular results from comparing canonical ensembles suggest that, while the Arg59-to-glutamine variation may modestly destabilize the RGS12 PDZ domain, it likely does not negatively impact, and may even slightly enhance, peptide-binding affinity, implying that this R59Q variation is unlikely to affect ligand selectivity severely.

### 2.5. SPR Testing of Ligand Binding to Wildtype and R59Q Variant RGS12 PDZ Domains

**Figure 7:**
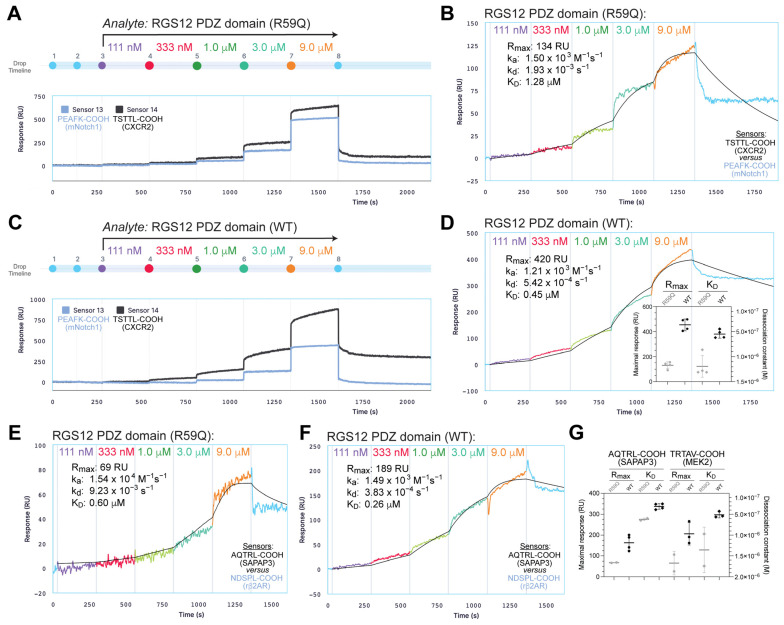
Ligand-binding affinities of R59Q-variant and wildtype RGS12 PDZ domain proteins, as measured by SPR biosensors. (**A**,**C**) Real-time measurements of optical response units (RUs) over time (in seconds) upon passing sequential drops of indicated concentrations of R59Q-variant RGS12 PDZ domain GST-fusion protein (panel **A**) or wildtype RGS12 PDZ domain GST-fusion protein (panel **C**) over streptavidin–gold nanosensors pre-bound with a negative control biotinylated peptide (mNotch1 C-tail; light blue) or biotinylated CXCR2 C-tail peptide TSTTL-COOH (black). (**B**,**D**) Test-surface SPR signal traces in panels (**A**) and (**C**), respectively, were subtracted from negative control surface traces to generate panels (**B**) and (**D**) prior to calculations of maximal binding (R_max_), apparent on-rate (k_a_), apparent off-rate (k_d_), and estimated dissociation constant (K_D_). Black line represents plot of idealized trace using 1:1 Langmuir kinetics model with mass transport limitation (Alto software version 2.3.1; Nicoya). (**D** inset), Plots of mean ± std. dev. for maximal response (R_max_ in RUs, circles; left *y*-axis) and for dissociation constant (K_D_ in molar, diamonds; right *y*-axis); N = 4 for both protein analytes interacting with CXCR2 TSTTL-COOH biosensor (R59Q variant in gray; wildtype in black). (**E**,**F**) Control surface-subtracted SPR signal traces for R59Q-variant RGS12 PDZ domain GST-fusion protein (panel **E**) or wildtype RGS12 PDZ domain GST-fusion protein (panel **F**) passed as analytes over streptavidin–gold nanosensors pre-bound with a negative control biotinylated peptide (rat beta2-adrenergic receptor C-tail) or biotinylated SAPAP3 C-tail peptide AQTRL-COOH. (**G**) Plots of mean ± std. dev. for maximal response (R_max_ in RUs, circles; left *y*-axis) and for dissociation constant (K_D_ in molar, diamonds; right *y*-axis); N = 2 for R59Q variant vs. SAPAP3 or MEK2 C-tail biosensors, N = 3 for wildtype PDZ domain vs. MEK2 C-tail biosensor, and N = 4 for wildtype PDZ domain vs. SAPAP3 C-tail biosensor.

To test whether the BD-associated R59Q variation affects the binding affinity of the human RGS12 PDZ domain, open-reading frames encoding the wildtype and R59Q-variant PDZ domains were separately and identically cloned as glutathione-S-transferase (GST) fusion proteins in bacterial expression plasmids, expressed in *E. coli*, and purified for in vitro testing (Supplementary Figure S5). Each of the three C-tail ligands was synthesized as an N-terminally biotin-tagged polypeptide and used to create gold nanoparticle biosensors for measuring the binding of these recombinant GST-RGS12 PDZ domain fusion proteins in real-time using surface plasmon resonance detection. Two additional biotinylated polypeptides corresponding to the C-termini of the mouse Notch1 and rat beta2-adrenergic receptor proteins, each previously shown to have no demonstrable affinity for the wildtype RGS12 PDZ domain in prior SPR assays [19,42,65], were also synthesized for creating negative control nanoparticle biosensors to run in parallel. For each of the three C-tail ligands tested (TSTTL-COOH of CXCR2, Figure 7A–D; TRTAV-COOH of MEK2 and AQTRL-COOH of SAPAP3; Figure 7E–G), the R59Q-variant PDZ domain fusion protein was observed to provide a less maximal binding signal (R_max_) in comparison to the wildtype PDZ protein and have an estimated dissociation constant (K_D_) of at least 2-fold less than the wildtype for each of the three peptide-laden biosensors (statistics summarized in graphs within Figure 7D,G).

### 2.6. MD Simulations of De Novo Structural Models Derived from AlphaFold2 (AF2)

**Figure 8:**
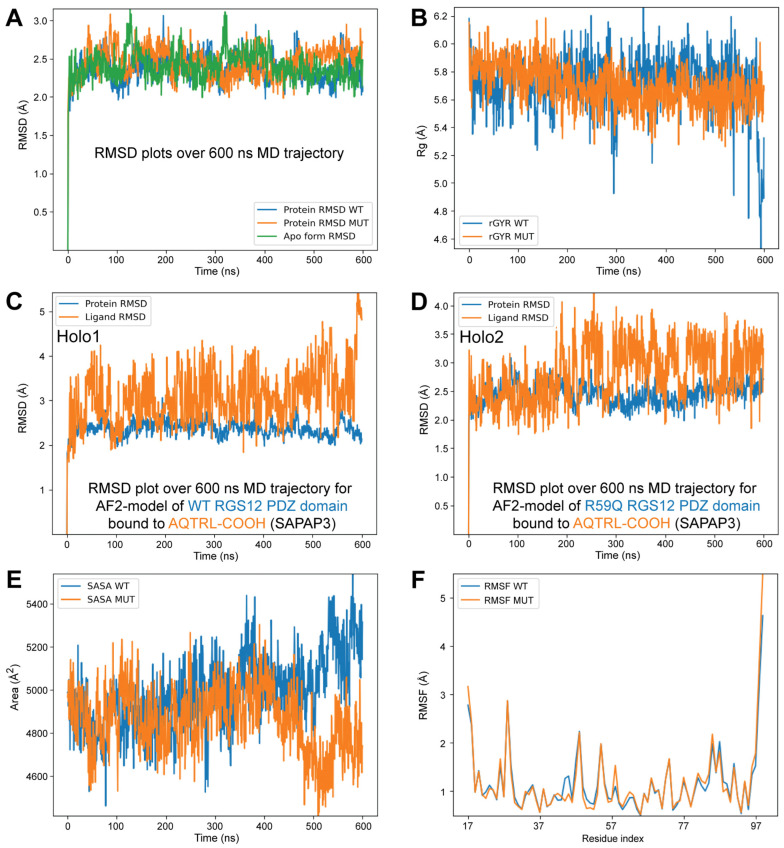
Representative results of MD simulations of unliganded and SAPAP3-liganded RGS12 PDZ domain models from AlphaFold2. (**A**) Plots of Cα-backbone root-mean-square deviation (RMSD; Å) over time (nanoseconds) during 600 ns MD simulation trajectories for the protein components of all three AF2-derived protein models (“Apo” = unliganded wildtype RGS12 PDZ domain; “WT” = Holo1 PDZ domain; “MUT” = Holo2 PDZ domain). (**B**) Plots of radius of gyration (rGyr or “Rg”; Å), for the AQTRL-COOH peptide ligand within the wildtype (“WT”) Holo1 complex and the R59Q-variant (“MUT”) Holo2 complex. (**C**,**D**) Plots of Cα-backbone RMSD over time during the MD trajectory for (panel **C**) PDZ domain (“protein”) and AQTRL-COOH ligand components of the wildtype Holo1 model, and for (panel **D**) PDZ domain (“protein”) and AQTRL-COOH ligand components of the R59Q-variant Holo2 model. (**E**) Solvent-accessible surface area (SASA; Å^2^) of the wildtype RGS12 PDZ domain (“WT” = Holo1 complex) or R59Q-variant PDZ domain (“MUT” = Holo2 complex) during the MD trajectories. (**F**) Root-mean-square fluctuation plots (RMSF; Å) for each residue of the wildtype ("WT") and R59Q-variant (“MUT”) RGS12 PDZ domain bound to AQTRL-COOH during the 600 ns MD simulation. RMSF values reflect the flexibility of each amino acid position over the course of the simulation, with larger RMSF values indicating higher atomic fluctuation. Both the wildtype and mutant PDZ domains exhibited regions of heightened flexibility, particularly at the N-terminal and C-terminal residues, although overall trends in flexibility were similar between the two models. Per-residue fluctuation was on average only 1.25% higher in Holo2.

In an attempt to resolve the apparent discrepancy between MD-based predictions of negligible change to slightly enhanced pentameric ligand-binding affinity by the R59Q-variant RGS12 PDZ domain versus the observed in vitro reductions in binding affinity in SPR-based biosensor assays (Figure 7), independent de novo structural models were created (using AlphaFold2; refs. [72,73]) of the unliganded and SAPAP3 C-tail liganded RGS12 PDZ domain (the latter with both wildtype and R59Q-variant sequences). The highest ranked of these new AF2 models were then solvated and input into MD simulations using a 600 ns relaxation-and-production trajectory similar to those previously described for the prior models based on PDB id 2KV8 NMR coordinates. Tracking Cα-backbone root-mean-square deviation (RMSD) across the 600 ns MD trajectories suggested that all three proteins undergo similar fluctuations. The unliganded AF2 model (“Apo”) stabilized at an average of 2.40 Å RMSD, and the wildtype SAPAP3-liganded PDZ model (“Holo1”) and the R59Q-variant SAPAP3-liganded model (“Holo2”) averaged 2.37 Å and 2.45 Å RMSD, respectively (Figure 8A). The overall compactness or “extendedness” of the AQTRL-COOH peptide ligand over the MD trajectories of both complexes was estimated by its radius of gyration (rGyr)—the principal moment of the ligand’s inertia. The SAPAP3 ligand’s rGyr variation in the receptor binding pocket of the wildtype RGS12 PDZ domain (Holo1) ranged from 4.5 Å to 6.3 Å, while the peptide ligand within Holo2 demonstrated rGyr values from 5.2 Å to 6.2 Å, suggesting little in the way of the differences predicted for this particular parameter (Figure 8B).

The protein RMSD for Holo1 ranged from 2 to 3 Å throughout the simulation time period. In the case of Holo2, the values for the protein RMSD values similarly ranged from 2 to 3 Å throughout the same period (Figure 8C,D). Tracking ligand RMSD in the same trajectories also revealed only minor differences overall. The ligand RMSD for the Holo1 complex ranged from 2 to 4 Å for the majority of the trajectory, only increasing beyond 4 Å after 500 ns (Figure 8C), whereas the ligand RMSD for Holo2 ranged from 2 to 3 Å for the first 200 ns, and then exhibited a wider range of 2 to 4 Å for the rest of the trajectory (Figure 8D). The solvent-accessible surface area (SASA) of each RGS12 PDZ domain was similarly fairly stable over the course of the trajectory (Figure 8E). The average SASA for the peptide-complexed wildtype RGS12 PDZ domain was 4983 Å^2^, whereas the average for the R59Q-variant PDZ domain was 4861 Å^2^.

**Figure 9:**
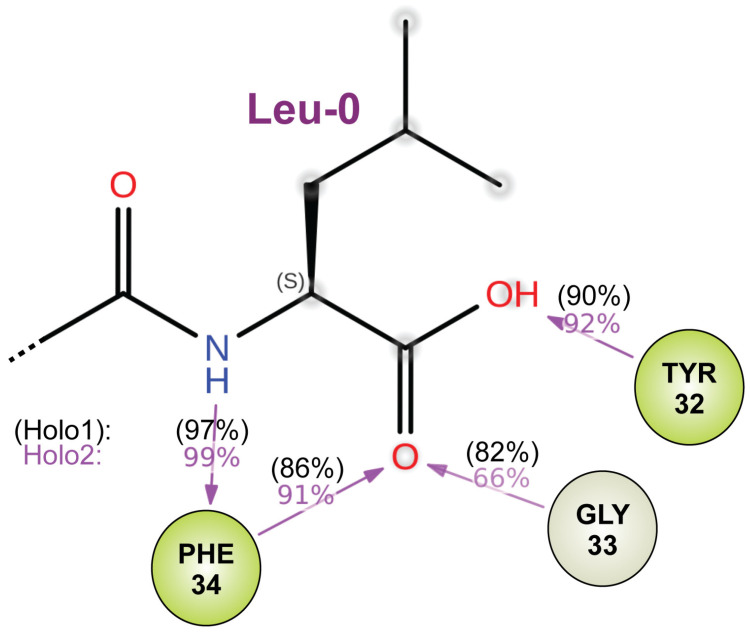
Predicted differences in hydrogen bond occupancy of the G-Φ-G-Φ motif during MD trajectory for elements of the final amino acid (“Leu-0”) and carboxylate moiety of the SAPAP3 ligand (AQTRL-COOH). Values obtained for the wildtype RGS12 PDZ domain model (Holo1) are in black brackets; values for the R59Q variant model (Holo2) are in magenta.

Root-mean-square fluctuation (RMSF) analysis provided insight into the flexibility of individual residues across the 600 ns MD simulations (Figure 8F), revealing fluctuations in protein dynamics not necessarily captured by overall RMSD or SASA measurements. The RMSF values for both the wildtype and R59Q-variant PDZ domains were generally low (around 1 to 2 Å), indicating relatively stable behavior. However, higher fluctuations were observed in the N- and C-terminal residues, with the RMSF values exceeding 4 Å for the latter, suggesting that these regions are more flexible, particularly in the R59Q variant. Quantifying the occupancy of the modeled hydrogen bond and the hydrophobic interactions between the specific residues of the AF2-modeled PDZ domains and the AQTRL-COOH pentameric ligand during the MD trajectories revealed per-residue interaction differences suggestive overall of weaker engagement of the ligand in the binding cleft of the R59Q variant. For example, as illustrated in Figure 9, the predicted interaction between the second glycine of the G-Φ-G-Φ motif (Gly-33) and the carbonyl oxygen of the ligand’s C-terminus decreased in overall persistence (66% occupancy) in the R59Q-variant PDZ domain versus 82% occupancy for the same hydrogen bonding in the wildtype PDZ domain model.

## 3. Discussion

Within both the established and predicted RGS12 PDZ domain structural models, its arginine-59 side chain is seen to be distant from its presumptive peptide-binding cleft, likely excluding any direct interaction from this amino acid position to a bound peptide ligand or cleft-defining residues. Our consistent observation in the SPR measurements of reduced binding affinity exhibited in vitro by the R59Q-variant PDZ domain is, therefore, suggestive of an indirect effect of this substitution on peptide-binding affinity and contrasts with our initial in silico molecular modeling, docking, and dynamics simulations that each predicted nil or slightly positive influences of the substituted glutamine side chain to the activity of the domain’s peptide-binding cleft. As another example of “negligible to slightly positive” predictions for the R59Q variation on PDZ function, the Schrödinger FEP+ MD simulation, first performed with the NMR-derived wildtype domain structure vs. its three C-tail targets and six alchemical permutations (Figure S4A), was also conducted with pose 16 of PDB id 2KV8 bearing the glutamine-59 substitution. The R59Q variation was again predicted to slightly improve the aggregated solvation-and-binding energy change upon formation of the TSTTL-COOH liganded complex (Figure S4D, summarized in Figure S4B). De novo models generated by AlphaFold2 also exhibited only minor differences in MD simulation trajectories. However, these differences appropriately trended toward predicting a reduced ligand-binding affinity for the R59Q-variant RGS12 PDZ domain (e.g., Figure 9).

Along this path to predicting, and then demonstrating, an effect of the R59Q variation on the PDZ domain binding function, we have reinforced that there are at least three candidate in vivo binding partners for the PDZ domain of human RGS12, namely, the C-termini of the proteins CXCR2, MEK2, and (now) SAPAP3:

**CXCR2**, a chemokine-family GPCR activated by interleukin-8 and primarily recognized for its role in immune regulation and inflammation [74,75], is also expressed in the microglia within the CNS [76]. As inflammation can significantly impact the pathophysiology of bipolar disorder [77], including alterations in microglial activity being linked to mood regulation and neuroplasticity [78], a change in the interaction between RGS12 and CXCR2, given the R59Q variation, may influence the microglial and/or global immune system responses, potentially affecting the cytokine profiles and neuronal signaling pathways crucial to the development and exacerbation of bipolar disorder symptoms (e.g., depression, as reviewed in [79]).
**MEK2** is integral to the MAPK/ERK signaling pathway [80,81], which is crucial for neuronal development, survival, and plasticity (e.g., ref. [82]). The interaction of RGS12 with MEK2, as we previously reported in identifying its involvement in TrkA/NGF signaling [42], may influence neurodevelopmental processes and synaptic plasticity (e.g., ref. [83]). Disruptions in this pathway have been implicated in the pathogenesis of various psychiatric disorders [84], including bipolar disorder [85], by affecting the neuronal circuitry and potentially contributing to the neurobiological underpinnings of mood dysregulation and cognitive impairments observed in the disorder.
**SAPAP3** is a synaptic scaffolding protein that interacts with postsynaptic density proteins like SHANK, playing a critical role in the structuring of synaptic junctions [59]. Mutations and dysfunctions in SAPAP3 and the associated proteins have been linked to neuropsychiatric disorders, most notably obsessive–compulsive disorder [59,86]. The interaction of RGS12 with SAPAP3 might affect synaptic stability and signaling, key areas of interest in bipolar disorder research focusing on synaptic homeostasis disruptions as a core element of the disease’s neuropsychiatric manifestations [87,88].

Along with the potential to directly disrupt the interaction with CXCR2, MEK2, and/or SAPAP3, the R59Q variation in RGS12 may also affect this multi-domain and multifunctional protein in its established CNS circuitry functions. By potentially altering RGS12’s normal regulatory functions that impact kappa opioid receptor (KOR) signaling to dopamine transporter expression and reuptake function (reviewed in [39]), the BD-associated R59Q variation (or, for that matter, the Q547H variation also discovered by Forstner et al. [10]) could significantly impact the neurobiological systems involved in stress responses and addiction mechanisms in individuals carrying this variant. Given the established roles of RGS12 in modulating KOR signaling (i.e., dampening G protein output but enhancing β-arrestin recruitment), particularly within the dopamine-rich environments of the ventral striatum [23,24], any disruption caused by a BD-associated missense mutation may exacerbate the dynorphin-mediated effects in stress responses. Normally, KOR activation by dynorphins leads to a reduction in dopamine release, which is part of the body’s natural response to stress and can result in dysphoric or depressive states [29,89]. Reduced function of RGS12 due to a BD-associated sequence variation may lead to enhanced KOR signaling, further decreasing dopamine availability and disrupting dopamine homeostasis. This alteration could potentiate the aversive states linked to stress and increase the vulnerability to addictive or repetitive behaviors [90]. Such changes in the balance of kappa opioid and dopaminergic signaling are particularly critical in the context of bipolar disorder, where dysregulations in these pathways can significantly influence mood states [91,92,93] and, thus, contribute to both the depressive and manic phases of the disorder. In this way, understanding how the R59Q variation within RGS12, which reduces its PDZ domain’s binding affinity, affects these neurochemical pathways could provide valuable insights into the pathophysiology of bipolar disorder and highlight new therapeutic targets. Therefore, in parallel with these investigations into the functional effects of BD-associated RGS12 missense mutations, we are also working towards establishing the “druggability” of this key KOR regulatory protein [94,95]. Future work will also complement the relative binding MD analyses performed here with umbrella sampling combined with the weighted histogram analysis method (WHAM; ref. [96]) to obtain a more detailed free-energy profile of the PDZ domain–peptide interactions along the reaction coordinates.

## 4. Methods

### 4.1. Domain Architecture and Phylogenetic Analyses

The polypeptide sequence of the longest human RGS12 isoform (UniProt O14924, a.k.a. RGS12_HUMAN [1447 aa]) was provided to the Simple Modular Architecture Research Tool (SMART ver. 9.0; smart.embl-heidelberg.de) protein domain annotation resource in its “genomic mode” [97]. The polypeptide sequences of the 75 mammalian RGS12 orthologues thereby identified were then used to generate a Newick-formatted tree (based on taxonomy from the U.S. National Center for Biotechnology Information [NCBI]) and an iTOL-formatted protein domains dataset for subsequent provision into the Interactive Tree of Life (iTOL v.6.9) webtool (itol.embl.de; ref. [98]). To probe the level of conservation of the arginine-59 position within human RGS12 across mammalian species, the polypeptide sequences of the PDZ domains of all 75 mammalian RGS12 orthologues were also provided in FASTA format to the NCBI Multiple Sequence Alignment Viewer (MSA v.1.25.0) to generate a multiple sequence alignment highlighting differences in amino-acid identity across amino acids 30 to 99 of the human RGS12 PDZ domain.

### 4.2. Creating Models of the Liganded Wildtype RGS12 PDZ Domain by Receptor Grid Docking

Using the sequence and structural similarity between the human RGS12 PDZ domain (aa 22–99 of UniProt O14924) and the first PDZ domain of human NHERF1 (aa 14–94 of UniProt O14745), the specific amino acids within NHERF1 observed to interact with the CXCR2 C-terminal tail (PDB id 4JL7: Y24–H29, H72, V76, I79, R80) were cross-identified within each of the 20 lowest-energy conformations of the un-liganded human RGS12 PDZ domain previously established by NMR (PDB id 2KV8: Y32–S37, H77, V81, I84, and G85). These 20 receptor grids derived from the 20 available RGS12 PDZ domain NMR structural models were then employed in the molecular docking of three-dimensional conformations of four C-tail polypeptide ligands (TSTTL-COOH from CXCR2, TRTAV-COOH from MEK2, AQTRL-COOH from SAPAP3, and PEAFK-COOH as an established negative control from the mouse Notch1 protein C-terminus [65], rendered with Schrödinger’s LigPrep and Epik7 algorithms [99]), using Schrödinger’s standard precision (SP) peptide and extra-precision (XP) GLIDE calculations [64,67] on an EXXACT Tensor workstation with 96 Intel Xeon Gold 5220R 2.20-GHz CPUs (EXXACT Corp., Fremont, CA, USA). The predicted PDZ domain–peptide complex structural models were visualized with Schrödinger’s Maestro (Schrödinger version 2023-4 on CentOS7).

### 4.3. MD Simulations of the Arg59 Residue Within a Solvated Model of the Wildtype RGS12 PDZ Domain

The structural models of multiple low-energy NMR conformations of the human RGS12 PDZ domain (PDB id 2KV8) were each first subjected to virtual aqueous solvation in 0.15 M NaCl using the System Builder solvation tool of Schrödinger’s Desmond molecular dynamics system (version 2023-4) [69]. An orthorhombic solvation volume was prepared with a simple point charge (SPC) explicit water model in such a way that the minimum distance between the protein surface and the solvent surface is 10 Å. Furthermore, the solvated system was neutralized by adding the counter ions Na^+^ and Cl^−^ to balance the net charge of the system. Prior to a production run of 200 nanoseconds in the isothermal-isobaric ensemble (i.e., “NPT” a.k.a. constant number of particles, constant pressure [101.325 kPa], and constant temperature [26.85 °C]), sequential steps of energy minimization, thermalization (from −263.15 °C to 26.85 °C), and equilibrations in canonical (NVT) and isothermal–isobaric (NPT) ensembles were performed using the OPLS4 force field [100] and an EXXACT Tensor workstation with Nvidia Tesla V100 32Gb GPUs (EXXACT Corp., Fremont, CA, USA):

100 picoseconds of Brownian dynamics NVT at −263.15 °C with restraints on solute-heavy atoms;
12 picoseconds NVT at −263.15 °C with restraints on solute-heavy atoms;
12 picoseconds NPT at −263.15 °C with restraints on solute-heavy atoms;
12 picoseconds NPT with restraints on solute-heavy atoms;
24 picoseconds NPT without restraints;
200 nanosecond NPT production run (sampled every 1.0 ns).

### 4.4. FEP+ MD of Binding Affinities Among Three C-Tail Ligands and Congeneric Pentamers

To predict the relative binding affinities of pentameric ligands for the RGS12 PDZ domain, a free-energy perturbation molecular dynamics simulation employing ‘alchemical’ single amino acid transformations between the three candidate PDZ domain-binding pentamers (TSTTL-COOH, TRTAV-COOH, and AQTRL-COOH) was performed with Schrödinger’s FEP+ calculations suite (2023-4) [68,70,101,102] using structural coordinates from pose 16 of PDB id 2KV8 initially bound to TSTTL-COOH (as derived from prior GLIDE XP docking). A 9-node/18-edge permutation map connecting the three candidate PDZ domain-binding pentamers (illustrated in Supplementary Figure S4A) was constructed. Each node’s pentameric polypeptide was provided in SMILES 2D configuration to Schrödinger’s LigPrep and Epik tools [99] for all-atom 3D representation and isomer optimization in the pH range of 7.4 ± 2.0, aligned to the configuration of TSTTL-COOH within pose 16 of the RGS12 PDZ domain (using Schrödinger’s MCS Docking Ligand Alignment tool in Schrödinger version 2023-4 on CentOS7) and then re-parameterized (as required) using Schrödinger’s Force Field Builder algorithm and OPLS4 [100] to provide any missing torsions. Solvation and relative free-energy binding calculations were requested from the FEP+ algorithm using inputs of the apo structure of the RGS12 PDZ domain (pose 16 of PDB id 2KV8; both wildtype sequence and R59Q variant) and the nine permutated pentamers’ aligned 3D conformations.

### 4.5. Transient Transfection and Co-Immunoprecipitation

Monolayer cultures of the immortalized human embryonic kidney cell line HEK293T (ATCC CRL-3216) were transfected at 75–90% confluence using FuGENE6 transfection reagent (Promega; Madison, WI, USA), according to the manufacturer’s instructions, with 0.5 μg of pcDNA3.1-Flag-SAPAP3 expression plasmid DNA and/or 0.5 μg of pcDNA3.1-HA-RGS12 expression plasmid DNA [103]. Forty-eight hours later, the transfected cells were lysed with ice-cold Triton-X lysis buffer (20 mM Tris-HCl pH 7.5, 150 mM NaCl, 1 mM EDTA, 1 mM EGTA, and 1% Triton X-100, supplemented with protease inhibitors). The lysates were clarified by centrifugation (16,000× *g*) and then incubated overnight at 4 °C with 1 mg of HA- or Flag-epitope tag-specific antibody followed by precipitation with protein-A/G beads (Santa Cruz Biotechnology; Dallas, TX, USA). The beads were washed 3 times with ice-cold lysis buffer and the proteins were eluted in Laemmli buffer. The samples were resolved on 4–12% precast SDS-polyacrylamide gels (Novex/Invitrogen; ThermoFisher Scientific, Waltham, MA, USA), transferred to nitrocellulose, and immunoblotted with horseradish peroxidase (HRP)-conjugated primary antibodies to HA- or Flag-epitope tags and visualized by ECL chemiluminescence (GE Healthcare; Chicago, IL, USA).

### 4.6. MD Simulations of RGS12 PDZ Domains to Predict Ligand Selectivity Changes

To predict the individual contributions of R59Q, A30P, and G31P amino-acid changes to the global fold and ligand-binding affinities of the human RGS12 PDZ domain, physics-based molecular dynamics free-energy perturbation simulations were conducted with the three different liganded structural models of pose 16 from PDB id 2KV8, as derived from receptor grid docking described above (i.e., first three columns of row 16 of Table 1), using Schrödinger’s FEP Protein Mutation for Ligand Selectivity panel (2023-4 version) with default settings [101]. FEP molecular dynamics simulations were run of single residue mutations within each liganded RGS12 PDZ domain model using an FEP+ workflow [70] and employing the Desmond molecular dynamics engine [69]. Each liganded RGS12 PDZ domain model was first solvated with 200 waters in the region around the single amino acid mutation or peptide-binding site. Enhanced water sampling was performed during the MD simulations via grand canonical Monte Carlo simulation [104]. Bennett’s approach [71] was used for estimating the free-energy difference between the canonical ensembles.

### 4.7. Surface Plasmon Resonance (SPR) Biosensor Measurements

#### 4.7.1. Single Concentration Analyses

N-terminally biotinylated peptides corresponding to the C-termini of mouse Notch1, CXCR2, and SAPAP3 were synthesized by Fmoc chemistry and purified by the Tufts University Core Facility (Dr. Michael Berne, director):

mNotch1, biotin-NH-PSQITHIPEAFK-carboxylic acid;
CXCR2, biotin-NH-PKDSRPSFVGSSSGHTSTTL-carboxylic acid;
SAPAP3 wt (aa 960-979), biotin-NH-SATESADSIEIYIPEAQTRL-carboxylic acid;
SAPAP3 mutant (“mt”), biotin-NH-SATESADSIEIYIPEAQSRL-amide
    (mutations underlined).

Optical detection of real-time protein–peptide interactions by surface plasmon resonance was performed using a Biacore 3000 (Cytiva; Marlborough, MA, USA), as per previously described methods [65,105,106]. Briefly, biotinylated peptides were separately immobilized on streptavidin-coated SPR chips (Cytiva) to 350 resonance units (RUs). All experiments were performed with the sensor surface, fluidics, and pump equilibrated with Biacore running buffer (10 mM HEPES pH 7.4, 250 mM NaCl, 0.05% (*v*/*v*) Nonidet P-40, and 50 μM EDTA). Increasing concentrations (10 nM to 500 μM) of recombinant RGS12 PDZ domain protein (expressed from *E. coli* culture and purified as previously described in ref. [65]) were separately injected at a flow rate of 10 μL/min with a 300 s dissociation phase. The surface was regenerated between each test concentration by the injection of a denaturing regeneration buffer (1 M NaCl, 50 mM NaOH). Non-specific binding to a biotinylated mNotch1 peptide-bound streptavidin surface was subtracted from each curve (BIA-evaluation Version 3.0 software, Cytiva). Maximal response units for each injection were plotted using GraphPad Prism 5.0.

#### 4.7.2. Single-Cycle Kinetics Analyses

Wildtype and R59Q-variant versions of the human RGS12 PDZ domain (aa 18–101 of UniProt O14924) were each cloned in frame with an N-terminal glutathione-S-transferase (GST) expression tag, expressed by *E. coli* fermentation, and purified by GST-affinity column and size-exclusion column chromatographies by GenScript. These recombinant GST-fusion proteins were stored at −80 °C in a storage buffer (150 mM NaCl, 10 mM Tris-HCl pH 7.6, 1 mM EDTA, 5 mM DTT, and 10% glycerol) prior to thawing and supplementation with 0.1% (final) Tween 20 for use in the SPR experiments. N-terminally biotinylated peptides corresponding to the C-termini of mouse Notch1, rat beta2-adrenergic receptor, CXCR2, MEK2, and SAPAP3 were synthesized and purified by GenScript:

mNotch1, biotin-Ahx-PSQITHIPEAFK-carboxylic acid;
rβ2AR, biotin-Ahx-QGRNCNTNDSPL-carboxylic acid;
CXCR2, biotin-Ahx-VGSSSGHTSTTL-carboxylic acid;
MEK2, biotin-Ahx-RTLRLKQPSTPTRTAV-carboxylic acid;
SAPAP3, biotin-Ahx-IYIPEAQTRL-carboxylic acid.

Optical detection of real-time protein–peptide interactions by surface plasmon resonance was performed using an Alto 8 × 2 digital microfluidics SPR device (Nicoya; Kitchener, ON, Canada). Biotinylated peptides were separately immobilized onto streptavidin-coated gold nanosensor particles (Nicoya) in lane pairs. The reference sensor of the lane held either immobilized mNotch1 or rβ2AR peptide as an established negative control surface [19,65], and the test sensor held one of the candidate PDZ domain-interacting peptides. All on-board dilutions and experimental passages of analytes over immobilized peptides were performed with a running buffer consisting of phosphate-buffered saline (137 mM NaCl, 2.7 mM KCl, 4.3 mM disodium phosphate, 1.4 mM monopotassium phosphate; and pH 7.3, from Teknova) supplemented with 0.1% Tween 20 (Nicoya), 1 mg (*w*/*v*) bovine serum albumin (BSA; Nicoya), and 3% glycerol (the latter to reduce bulk-shift readings when transitioning from running buffer to protein analyte injections). “Single-stack” (single-cycle) kinetic analyses were performed by the serial passage of 3-fold-increasing concentrations (111 nM–9 μM) of purified, recombinant GST-RGS12 PDZ domain fusion protein, followed by a dissociation phase of running buffer passage. Each sensor was regenerated between each single-stack passage by the injection of a surface regeneration buffer (10 mM NaOH). Non-specific binding to the paired, negative control peptide-bound streptavidin surface was subtracted from each single-stack curve and then binding parameters of the on-rate (k_a_), off-rate (k_d_), and dissociation constant (K_D_) were calculated using the 1:1 Langmuir kinetics model with mass transport limitation (Alto software version 2.3.1; Nicoya). Observed maximal response units (R_max_) and calculated K_D_ values for each single-stack injection were plotted using GraphPad Prism 10.

### 4.8. Creating De Novo Models of Unliganded and Liganded RGS12 PDZ Domain with AlphaFold2

Three-dimensional structures of the unliganded wildtype RGS12 PDZ domain (“Apo”), wildtype RGS12 PDZ domain bound to SAPAP3’s AQTRL-COOH (“Holo1”), and R59Q-variant RGS12 PDZ domain bound to SAPAP3’s AQTRL-COOH (“Holo2”) were each predicted using the AlphaFold2 (AF2 v.1.5.5) algorithm, as implemented on the Colab server [73] and accessed on 12 February 2024 (https://colab.research.google.com/github/sokrypton/ColabFold/blob/main/AlphaFold2.ipynb#scrollTo=G4yBrceuFbf3). Unlike our prior models described above that were templated on NMR-established structural coordinates within PDB id 2KV8, each AF2 prediction was performed without considering any homologous experimental template (“template mode: none”) and with three as the number of recycles. For each structural model, the best-predicted model (rank 1) out of the five computed by AF2 was taken into the MD simulations (described below). A reliability ranking of the AF2 predictions was performed using the local distance difference test (LDDT) score and the predicted aligned error (PAE) matrices reported for each AF2-generated structural model.

### 4.9. MD Simulations of AlphaFold2-Derived Structural Models

AF2-generated structural models were each protonated using Schrödinger’s Protein Preparation wizard [107]. Then, each was subjected to a 600 ns MD simulation production run as previously described above (see Methods Section 4.3) using Schrödinger Release version 2024-1. Predicted structural changes during the 600 ns MD trajectory, including backbone root-mean-square deviation (RMSD) and per-residue root-mean-square fluctuation (RMSF), the radius of gyration (Rg), solvent-accessible surface area (SASA), and quantitation of protein–ligand interaction occupancies, were subsequently obtained using Schrödinger’s simulation interaction diagram (SID) wizard [69].

## 5. Conclusions

While the relative position of the BD-associated R59Q variation excludes direct contact between the 59th amino acid’s side chain and the RGS12 PDZ domain’s ligand or its ligand-binding site, the substitution of glutamine for arginine at this position is observed to undermine the full binding function of the PDZ domain in vitro. These observations of reduced binding in vitro suggest that the replacement of arginine with glutamine in BD probands may diminish RGS12’s binding strength to its in vivo targets, including the candidate interactors CXCR2, MEK2, and SAPAP3. Such a reduction in functional performance could negatively influence central nervous system signaling pathways, specifically those involving dynorphin and dopamine, which could potentially exacerbate the pathophysiology of bipolar disorder in affected individuals.

## Figures and Tables

**Table 1:** Heat map of GLIDE SP-peptide and GLIDE XP scores for predicted docking of four pentameric ligands into the ligand-binding “receptor grid” specified for each of the 20 low-energy structural models (“poses”) of the human RGS12 PDZ domain recorded within PDB id 2KV8. GLIDE SPpep and XP scores are indexed similarly to Gibbs’ free energy but do not explicitly use kcal/mol units; a more negative value indicates that tighter binding is predicted. Ligands used in docking include the CXCR2 C-tail TSTTL-COOH, the MEK2 C-tail TRTAV-COOH, the SAPAP3 C-tail AQTRL-COOH, and a negative control derived from the C-terminus of the mouse Notch1 protein (PEAFK-COOH) previously established to have no demonstrable binding to recombinant RGS12 PDZ protein [19,42,65]. Note that pose 16 provides the darkest green shading for each GLIDE score, except for the negative control peptide.

2KV8 Pose	Ligand	GLIDE SPpep Score	GLIDE XP Score	Ligand	GLIDE SPpep Score	GLIDE XP Score	Ligand	GLIDE Sppep Score	GLIDE XP Score	Ligand	GLIDE SPpep Score	GLIDE XP Score
**1**	TSTTL-COOH (CXCR2)	−5.5	−7.8	TRTAV-COOH (MEK2)	−6.8	−7.2	AQTRL-COOH (SAPAP3)	−6.4	−5.5	PEAFK-COOH (mNotch1)	−6.7	−7.3
**2**	TSTTL-COOH (CXCR2)	−6.1	−8.0	TRTAV-COOH (MEK2)	−5.2	−7.9	AQTRL-COOH (SAPAP3)	−6.2	−7.0	PEAFK-COOH (mNotch1)	−6.9	−5.6
**3**	TSTTL-COOH (CXCR2)	−5.9	−7.7	TRTAV-COOH (MEK2)	−5.9	−7.2	AQTRL-COOH (SAPAP3)	−6.1	−6.5	PEAFK-COOH (mNotch1)	−5.4	−5.4
**4**	TSTTL-COOH (CXCR2)	−5.7	−6.8	TRTAV-COOH (MEK2)	−5.9	−6.5	AQTRL-COOH (SAPAP3)	−5.4	−6.6	PEAFK-COOH (mNotch1)	−6.2	−4.9
**5**	TSTTL-COOH (CXCR2)	−6.2	−9.0	TRTAV-COOH (MEK2)	−6.0	−7.7	AQTRL-COOH (SAPAP3)	−5.8	−7.8	PEAFK-COOH (mNotch1)	−6.3	−5.7
**6**	TSTTL-COOH (CXCR2)	−7.1	−8.5	TRTAV-COOH (MEK2)	−5.6	−7.1	AQTRL-COOH (SAPAP3)	−7.9	−8.0	PEAFK-COOH (mNotch1)	−8.1	−6.4
**7**	TSTTL-COOH (CXCR2)	−6.7	−8.3	TRTAV-COOH (MEK2)	−6.6	−8.0	AQTRL-COOH (SAPAP3)	−6.5	−7.3	PEAFK-COOH (mNotch1)	−6.8	−6.5
**8**	TSTTL-COOH (CXCR2)	−6.9	−8.5	TRTAV-COOH (MEK2)	−6.1	−7.7	AQTRL-COOH (SAPAP3)	−7.9	−8.4	PEAFK-COOH (mNotch1)	−8.1	−6.1
**9**	TSTTL-COOH (CXCR2)	−7.2	−7.6	TRTAV-COOH (MEK2)	−4.9	−6.4	AQTRL-COOH (SAPAP3)	−5.6	−6.2	PEAFK-COOH (mNotch1)	−6.2	−5.8
**10**	TSTTL-COOH (CXCR2)	−6.5	−9.7	TRTAV-COOH (MEK2)	−6.0	−7.6	AQTRL-COOH (SAPAP3)	−5.3	−6.7	PEAFK-COOH (mNotch1)	−7.1	−5.9
**11**	TSTTL-COOH (CXCR2)	−6.9	−9.5	TRTAV-COOH (MEK2)	−5.9	−9.3	AQTRL-COOH (SAPAP3)	−8.2	−9.2	PEAFK-COOH (mNotch1)	−7.7	−7.0
**12**	TSTTL-COOH (CXCR2)	−6.4	−8.4	TRTAV-COOH (MEK2)	−5.3	−7.1	AQTRL-COOH (SAPAP3)	−7.7	−8.4	PEAFK-COOH (mNotch1)	−5.9	−5.0
**13**	TSTTL-COOH (CXCR2)	−6.1	−8.2	TRTAV-COOH (MEK2)	−5.8	−7.8	AQTRL-COOH (SAPAP3)	−6.4	−6.8	PEAFK-COOH (mNotch1)	−6.4	−5.7
**14**	TSTTL-COOH (CXCR2)	−5.8	−7.9	TRTAV-COOH (MEK2)	−5.1	−7.0	AQTRL-COOH (SAPAP3)	−5.5	−6.8	PEAFK-COOH (mNotch1)	−6.6	−6.3
**15**	TSTTL-COOH (CXCR2)	−5.6	−8.2	TRTAV-COOH (MEK2)	−6.0	−6.4	AQTRL-COOH (SAPAP3)	−5.5	−7.2	PEAFK-COOH (mNotch1)	−7.3	−6.7
**16**	TSTTL-COOH (CXCR2)	−7.8	−12.4	TRTAV-COOH (MEK2)	−7.6	−10.8	AQTRL-COOH (SAPAP3)	−8.8	−12.1	PEAFK-COOH (mNotch1)	−7.2	−7.8
**17**	TSTTL-COOH (CXCR2)	−6.9	−8.5	TRTAV-COOH (MEK2)	−5.7	−8.0	AQTRL-COOH (SAPAP3)	−7.6	−7.8	PEAFK-COOH (mNotch1)	−5.6	−5.0
**18**	TSTTL-COOH (CXCR2)	−6.6	−7.9	TRTAV-COOH (MEK2)	−5.3	−7.7	AQTRL-COOH (SAPAP3)	−6.6	−8.4	PEAFK-COOH (mNotch1)	−7.1	−6.5
**19**	TSTTL-COOH (CXCR2)	−7.1	−8.6	TRTAV-COOH (MEK2)	−6.5	−5.9	AQTRL-COOH (SAPAP3)	−7.4	−7.3	PEAFK-COOH (mNotch1)	−5.4	−6.5
**20**	TSTTL-COOH (CXCR2)	−6.9	−7.7	TRTAV-COOH (MEK2)	−5.4	−6.8	AQTRL-COOH (SAPAP3)	−7.5	−7.4	PEAFK-COOH (mNotch1)	−7.2	−6.4
	average docking score:	−6.5	−8.5	average docking score:	−5.9	−7.5	average docking score:	−6.7	−7.6	average docking score:	−6.7	−6.1

**Table 2:** Comparison of best docking scores and MD calculated free-energy changes for indicated RGS12 PDZ domain–peptide complexes.

Peptide Within 2KV8 Pose 16	Best GLIDE SPpep Docking Score	Best GLIDE XP Docking Score	FEP+ Solvation + Binding Energy (ΔG; kcal/mol)
TSTTL-COOH (CXCR2)	−7.8	−12.4	−12.3 ± 0.5
TRTAV-COOH (MEK2)	−7.6	−10.8	−11.1 ± 0.4
AQTRL-COOH (SAPAP3)	−8.8	−12.1	−12.7 ± 0.4

## Data Availability

Structural coordinates for generated models will be provided upon reasonable request by email.

## Supplementary Materials

The following supporting information can be downloaded at: https://www.mdpi.com/article/10.3390/ijms252111431/s1.
