# Supplementary material for: Molecular Modeling and In Vitro Functional Analysis of the RGS12 PDZ Domain Variant Associated with High-Penetrance Familial Bipolar Disorder
Source: Int J Mol Sci. 2024 Oct 24;25(21):11431. doi: 10.3390/ijms252111431 (PMC11546610; doi:10.3390/ijms252111431)
Supplement: Supplementary file 1 [file ijms-25-11431-s001.zip › ijms-3163573-supplementary.pdf]

Supplementary Information

Molecular Modeling and In Vitro Functional Analysis of the RGS12 PDZ Domain Variant Associated with High-Penetrance Familial Bipolar Disorder

Percy S. Agogo-Mawuli [1], Joseph Mendez [1], Emily A. Oestreich [2],
Dustin E. Bosch [3] and David P. Siderovski [1,*]

* Correspondence:
David P. Siderovski
david.siderovski@unthsc.edu

[1] Department of Pharmacology & Neuroscience, University of North Texas Health Science Center, Fort Worth, TX 76107, USA

[2] Department of Biomedical Sciences, Pacific Northwest University of Health Sciences, Yakima, WA 98901, USA

[3] Department of Pathology, Carver College of Medicine, University of Iowa, Iowa City, IA 52242, USA

Figure S1

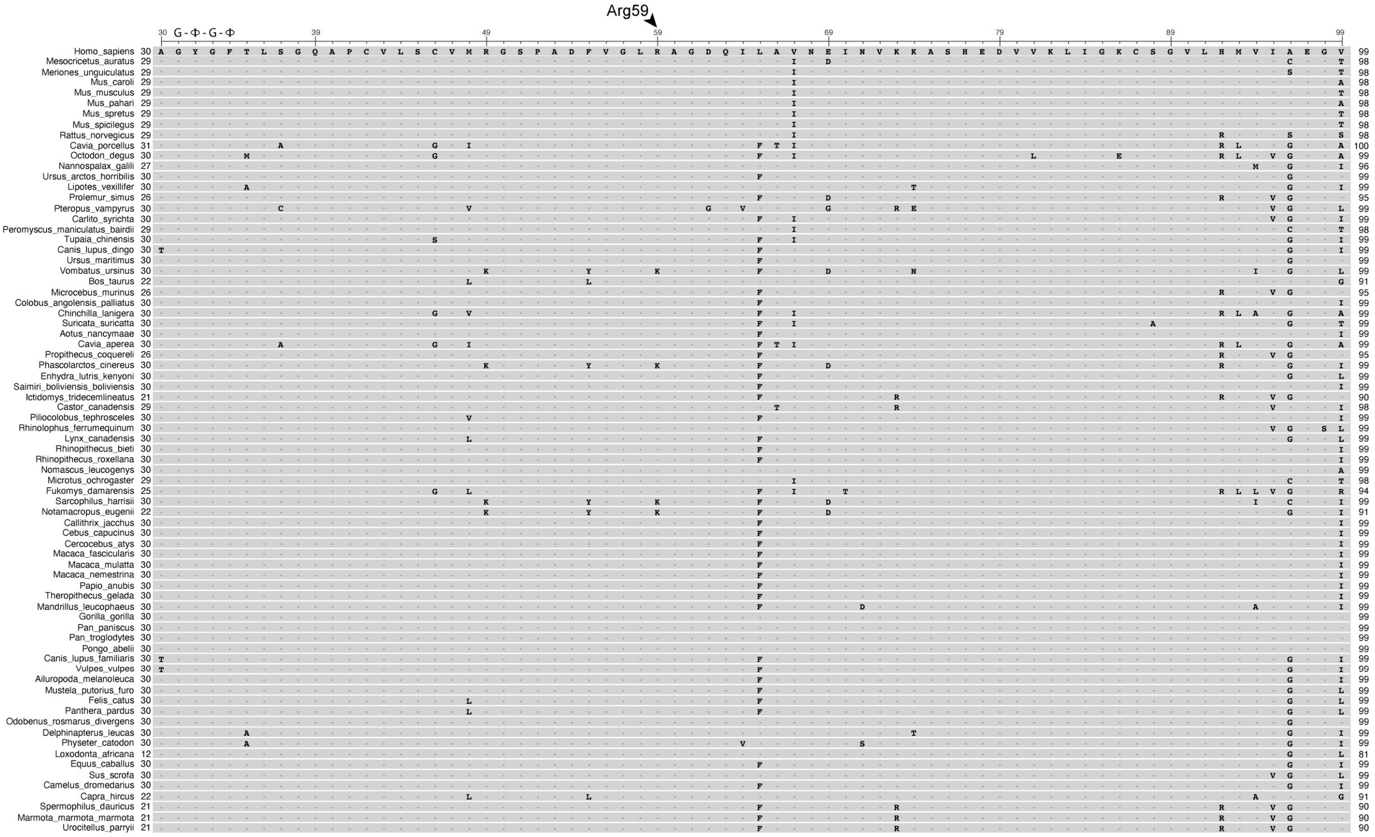

Fig. S1. Multiple sequence alignment (MSA) of PDZ domain sequences from 75 mammalian RGS12 orthologues. The PDZ domain sequence of human RGS12 (amino acids 30 to 99 of UniProt O14924) is displayed on the top row of the MSA. Subsequent rows contain the remaining 74 mammalian RGS12 orthologues as indicated by their species names. Identical amino acid residues within the non-human RGS12 orthologues are indicated by gray dots; only variations in their PDZ domain sequences are provided within the MSA. The G-Φ-G-Φ motif present in the human RGS12 PDZ domain sequence (Gly31 to Phe34) is completely conserved amongst all mammalian RGS12 orthologues. Similarly, the arginine residue (Arg59) that corresponds to the BD-associated R59Q variation in human RGS12 is highly conserved amongst most mammalian orthologues; however, four mammalian species diverge: a clade of Australian marsupials (*Vombatus ursinus, Phascolarctos cinereus, Sarcophilus harrisii,* and *Notamacropus eugenii)* have a different basic amino acid (K, lysine) present at the position corresponding to Arg59 of human RGS12.

Figure S2

A

2KV8 pose	1	2	3	4	5	6	7	8	9	10	11	12	13	14	15	16	17	18	19	20
1	0.00	1.64	1.04	1.15	1.86	1.93	1.15	1.54	1.38	1.25	1.36	1.76	1.28	1.05	1.27	2.05	1.89	1.42	1.93	2.22
2	1.64	0.00	1.41	1.44	1.41	1.36	1.61	1.45	1.20	1.47	1.46	1.40	1.30	1.50	1.40	1.60	1.61	1.29	1.44	1.53
3	1.04	1.41	0.00	0.99	1.91	1.61	1.22	1.41	1.17	1.27	1.42	1.62	0.95	1.14	1.19	1.97	1.97	1.27	1.78	2.18
4	1.15	1.44	0.99	0.00	1.93	1.68	1.30	1.54	1.19	1.35	1.44	1.68	1.12	1.20	1.20	2.20	2.03	1.42	1.90	2.16
5	1.86	1.41	1.91	1.93	0.00	2.01	1.83	2.07	1.94	1.93	1.64	1.80	2.02	1.74	1.90	1.45	1.26	1.73	1.43	1.12
6	1.93	1.36	1.61	1.68	2.01	0.00	1.82	1.35	1.47	1.81	2.03	1.08	1.52	1.97	1.75	1.93	2.06	1.48	1.68	1.80
7	1.15	1.61	1.22	1.30	1.83	1.82	0.00	1.48	1.65	1.00	1.12	1.56	1.27	0.97	1.11	1.67	1.43	1.47	1.99	2.09
8	1.54	1.45	1.41	1.54	2.07	1.35	1.48	0.00	1.50	1.29	1.54	1.09	1.20	1.46	1.14	1.80	1.89	1.37	1.51	2.18
9	1.38	1.20	1.17	1.19	1.94	1.47	1.65	1.50	0.00	1.52	1.77	1.67	1.12	1.54	1.56	2.18	2.17	1.20	1.78	2.07
10	1.25	1.47	1.27	1.35	1.93	1.81	1.00	1.29	1.52	0.00	1.10	1.58	1.18	1.10	0.91	1.78	1.68	1.45	1.87	2.29
11	1.36	1.46	1.42	1.44	1.64	2.03	1.12	1.54	1.77	1.10	0.00	1.60	1.45	0.92	0.98	1.57	1.42	1.73	1.77	2.13
12	1.76	1.40	1.62	1.68	1.80	1.08	1.56	1.09	1.67	1.58	1.60	0.00	1.41	1.65	1.37	1.61	1.76	1.44	1.31	1.79
13	1.28	1.30	0.95	1.12	2.02	1.52	1.27	1.20	1.12	1.18	1.45	1.41	0.00	1.25	1.06	1.91	1.96	1.26	1.67	2.15
14	1.05	1.50	1.14	1.20	1.74	1.97	0.97	1.46	1.54	1.10	0.92	1.65	1.25	0.00	1.01	1.77	1.58	1.45	1.77	2.15
15	1.27	1.40	1.19	1.20	1.90	1.75	1.11	1.14	1.56	0.91	0.98	1.37	1.06	1.01	0.00	1.73	1.69	1.46	1.72	2.26
16	2.05	1.60	1.97	2.20	1.45	1.93	1.67	1.80	2.18	1.78	1.57	1.61	1.91	1.77	1.73	0.00	1.02	1.98	1.74	1.60
17	1.89	1.61	1.97	2.03	1.26	2.06	1.43	1.89	2.17	1.68	1.42	1.76	1.96	1.58	1.69	1.02	0.00	1.94	1.80	1.50
18	1.42	1.29	1.27	1.42	1.73	1.48	1.47	1.37	1.20	1.45	1.73	1.44	1.26	1.45	1.46	1.98	1.94	0.00	1.50	1.92
19	1.93	1.44	1.78	1.90	1.43	1.68	1.99	1.51	1.78	1.87	1.77	1.31	1.67	1.77	1.72	1.74	1.80	1.50	0.00	1.68
20	2.22	1.53	2.18	2.16	1.12	1.80	2.09	2.18	2.07	2.29	2.13	1.79	2.15	2.15	2.26	1.60	1.50	1.92	1.68	0.00

B

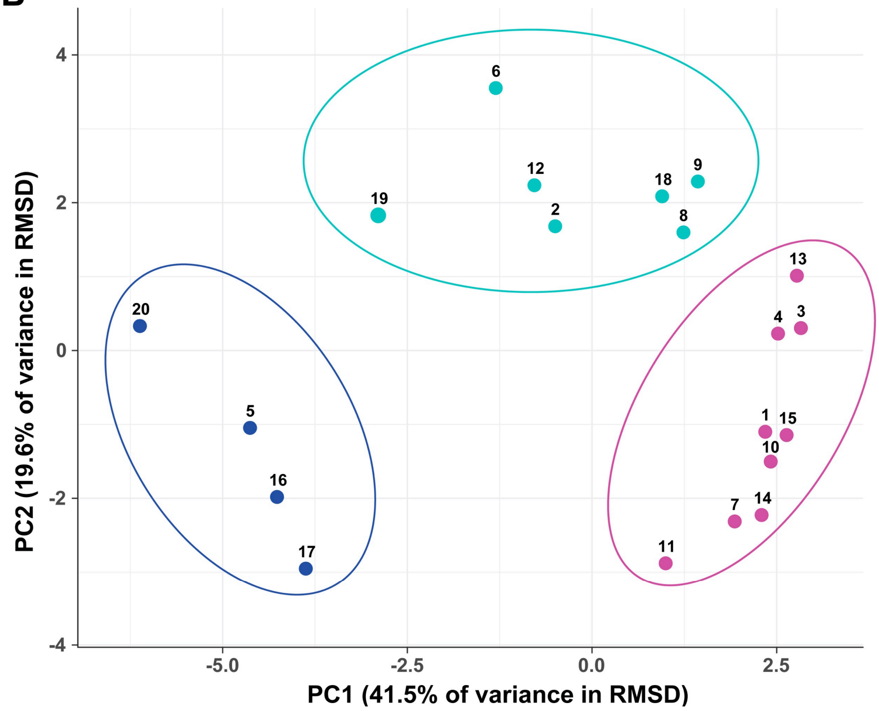

Fig. S2. (**A**) Heatmap displaying pairwise backbone root mean square deviation (RMSD) values between each of the 20 NMR-derived structural models of the unliganded RGS12 PDZ domain (PDB id: 2KV8). The RMSD values, measured in angstroms (Å), were calculated to assess structural variability among the models. Each cell in the heatmap represents the RMSD between two specific models, color-coded from green (low RMSD, indicating greater structural similarity) to red (high RMSD, indicating greater structural divergence). Visual Molecular Dynamics (VMD) software (https://www.ks.uiuc.edu/Research/vmd/) was used for RMSD calculations, with the diagonal showing each model's self-RMSD (0.00 Å; dark green). (**B**) Principal Component Analysis (PCA) plot illustrating the clustering of the 20 RGS12 PDZ domain structures based on their pairwise RMSD values. Unit variance scaling was applied to normalize the data, ensuring equal weighting of each variable. The analysis was conducted using singular value decomposition (SVD) to handle any missing data. The x-axis (PC1) explains 41.5% of the total variance, while the y-axis (PC2) accounts for an additional 19.6% of the variance. The 20 models are represented as individual points, with clusters of conformations visually differentiated by distinct colors (royal blue, teal, and magenta), reflecting structural similarity. ClustVis (https://biit.cs.ut.ee/clustvis/) was used for PCA and plotting.

Figure S3

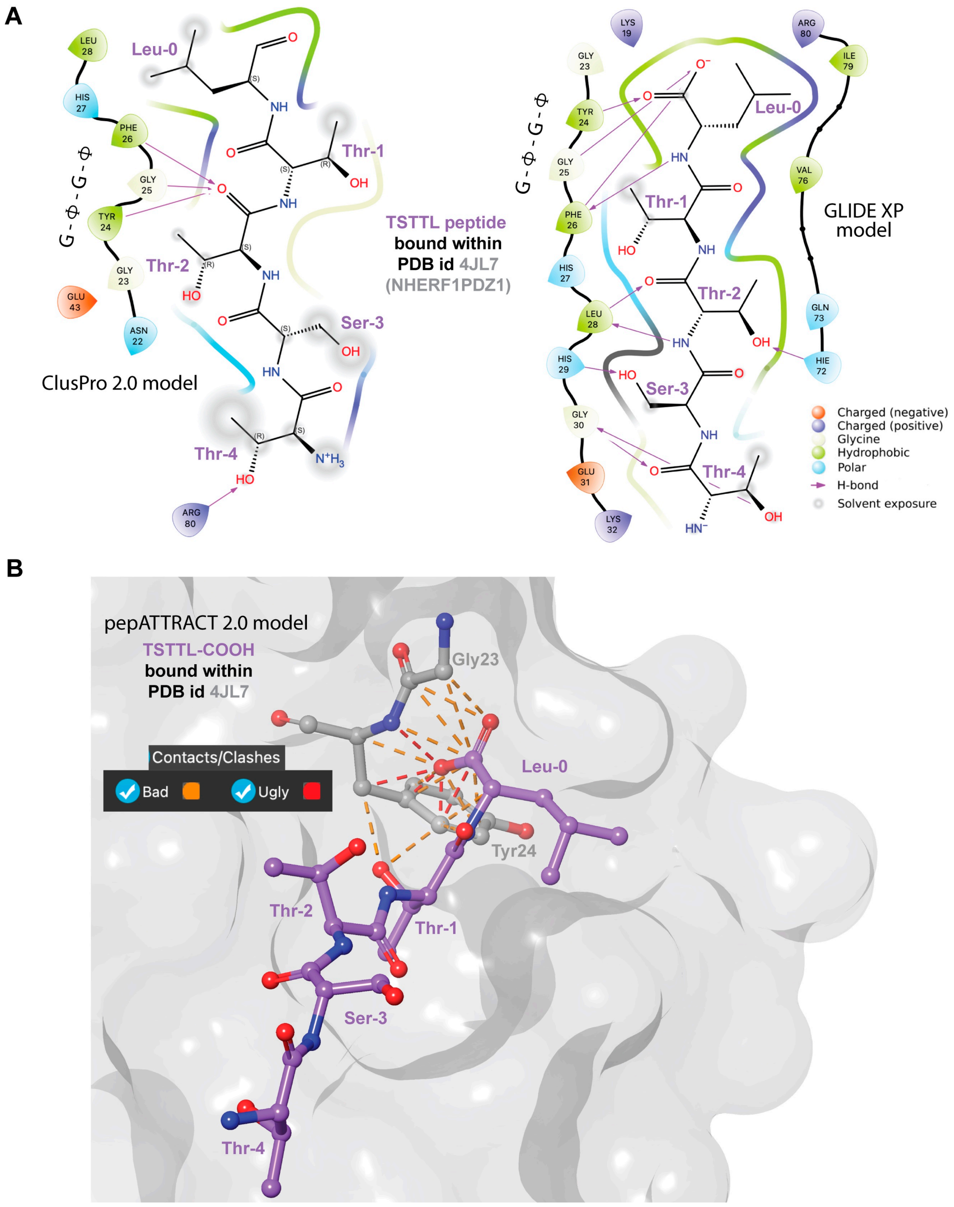

Fig. S3. Comparison of three protein/peptide docking algorithms -- ClusPro 2.0, GLIDE XP, and pepATTRACT 2.0 -- demonstrating differing predictions for the known NHERF1 PDZ1 domain interaction with the TSTTL-COOH terminal peptide from CXCR2 (PDB id 4JL7). (**A**) Schematic representations of predictions from ClusPro 2.0 (*left*) and GLIDE XP (*right*). In the ClusPro model, the TSTTL-COOH peptide adopts an orientation violating known PDZ domain/peptide interaction principles, particularly the improper alignment of the G-Φ-G-Φ motif with the peptide bond between Thr-2 and Thr-1. By contrast, the GLIDE XP model adheres to established PDZ domain/C-terminal peptide interaction principles, particularly the G-Φ-G-Φ motif's correct engagement with the peptide C-terminus and Leu-0. (**B**) Structural model of the TSTTL-COOH peptide bound to the NHERF1 PDZ1 domain (gray surface and residues Gly-23 and Tyr-24 of the G-Φ-G-Φ motif) as predicted by pepATTRACT 2.0. The molecular visualization highlights many steric clashes around the Leu-0 and Thr-1 residues of the bound peptide, as identified by Schrödinger's Maestro that quantitatively categorizes "bad" contacts (orange) and "ugly" contacts (red) based on the degree of atomic overlap between the peptide and the binding pocket. "Bad" contacts indicate mild steric clashes with less severe atomic overlaps, while "ugly" contacts represent more significant steric hindrances, where the overlap is too large for favorable interaction or binding. The GLIDE XP model, in contrast, shows no such clashes and aligns well with the canonical interaction framework for PDZ domains engaging C-tail peptides.

Figure S4

A

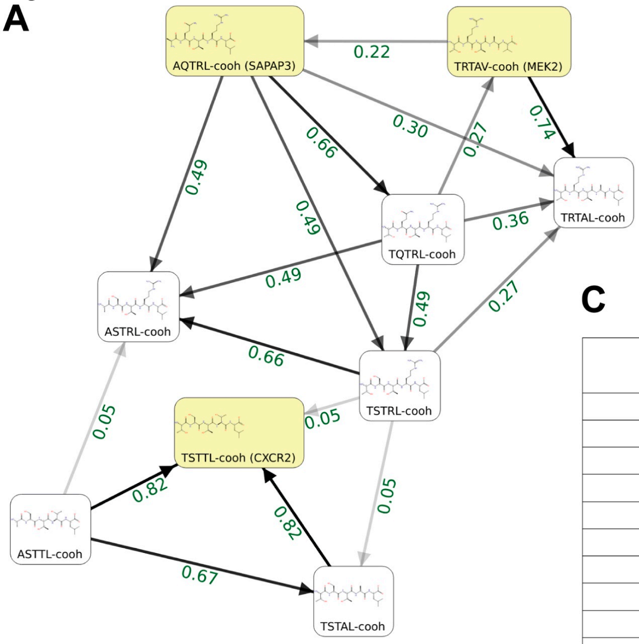

B

Sequence of RGS12 PDZ domain:	WT	WT	R59Q
**Peptide within 2KV8 pose 16**	**Best GLIDE XP docking score**	**FEP+ solvation + binding energy** (ΔG; kcal/mol)	**FEP+ solvation + binding energy** (ΔG; kcal/mol)
**TSTTL-cooh (CXCR2)**	-12.4	-12.3 ± 0.5	-13.1 ± 0.5
**TRTAV-cooh (MEK2)**	-10.8	-11.1 ± 0.4	-10.7 ± 0.5
**AQTRL-cooh (SAPAP3)**	-12.1	-12.7 ± 0.4	-12.7 ± 0.5

C

Peptide ligand bound in wildtype RGS12 PDZ domain (PDB id 2KV8 pose 16)

**Edge title** (node → node)	**Complex energy** (ΔG; kcal/mol)	**Solvent energy** (ΔG; kcal/mol)	Bennett error (ΔΔG; kcal/mol)	Cycle closure error (ΔΔG; kcal/mol)
TQTRL-cooh → TSTRL-cooh	55.65 ± 0.08	54.49 ± 0.08	1.16 ± 0.11	0.86 ± 0.96
TQTRL-cooh → TRTAV-cooh (MEK2)	47.63 ± 0.17	46.33 ± 0.14	1.30 ± 0.22	1.53 ± 0.95
TQTRL-cooh → TRTAL-cooh	56.42 ± 0.12	54.65 ± 0.09	1.76 ± 0.16	1.79 ± 0.73
TQTRL-cooh → ASTRL-cooh	47.78 ± 0.17	46.68 ± 0.16	1.10 ± 0.23	1.11 ± 0.91
TSTRL-cooh → TSTAL-cooh	3.38 ± 0.13	2.42 ± 0.08	0.96 ± 0.15	0.91 ± 0.73
TSTRL-cooh → TRTAL-cooh	3.06 ± 0.19	1.20 ± 0.17	1.86 ± 0.26	0.93 ± 1.23
TSTRL-cooh → ASTRL-cooh	-8.04 ± 0.10	-8.36 ± 0.06	0.32 ± 0.12	0.25 ± 0.83
TSTRL-cooh → TSTTL-cooh (CXCR2)	9.83 ± 0.13	9.38 ± 0.08	0.45 ± 0.16	0.67 ± 0.97
AQTRL-cooh (SAPAP3) → TQTRL-cooh	0.71 ± 0.07	0.63 ± 0.06	0.09 ± 0.09	0.06 ± 0.96
AQTRL-cooh (SAPAP3) → TSTRL-cooh	56.94 ± 0.09	55.49 ± 0.06	1.45 ± 0.11	0.92 ± 1.23
AQTRL-cooh (SAPAP3) → TRTAL-cooh	56.78 ± 0.16	55.60 ± 0.12	1.18 ± 0.20	1.85 ± 1.23
AQTRL-cooh (SAPAP3) → ASTRL-cooh	48.35 ± 0.09	47.06 ± 0.08	1.30 ± 0.12	1.17 ± 0.97
ASTTL-cooh → TSTAL-cooh	-2.33 ± 0.10	-2.65 ± 0.07	0.32 ± 0.13	0.39 ± 0.73
ASTTL-cooh → ASTRL-cooh	-147.41 ± 0.15	-146.96 ± 0.10	-0.45 ± 0.18	-0.27 ± 0.97
ASTTL-cooh → TSTTL-cooh (CXCR2)	4.59 ± 0.13	4.20 ± 0.08	0.40 ± 0.15	0.15 ± 0.97
TSTAL-cooh → TSTTL-cooh (CXCR2)	6.50 ± 0.11	6.76 ± 0.06	-0.26 ± 0.13	-0.24 ± 0.21
TRTAV-cooh (MEK2) → AQTRL-cooh (SAPAP3)	-48.76 ± 0.21	-47.18 ± 0.18	-1.57 ± 0.27	-1.59 ± 0.95
TRTAV-cooh (MEK2) → TRTAL-cooh	7.71 ± 0.10	7.70 ± 0.06	0.01 ± 0.12	0.26 ± 0.87

D

Peptide ligand bound in R59Q-variant RGS12 PDZ domain (PDB id 2KV8 pose 16)

**Edge title** (node → node)	**Complex energy** (ΔG; kcal/mol)	**Solvent energy** (ΔG; kcal/mol)	Bennett error (ΔΔG; kcal/mol)	Cycle closure error (ΔΔG; kcal/mol)
TQTRL-cooh → TSTRL-cooh	55.56 ± 0.10	54.36 ± 0.08	1.19 ± 0.13	1.04 ± 1.03
TQTRL-cooh → TRTAV-cooh (MEK2)	48.82 ± 0.20	46.57 ± 0.13	2.26 ± 0.24	2.00 ± 1.05
TQTRL-cooh → TRTAL-cooh	56.04 ± 0.12	54.37 ± 0.09	1.67 ± 0.15	2.03 ± 0.99
TQTRL-cooh → ASTRL-cooh	48.19 ± 0.19	46.75 ± 0.11	1.44 ± 0.22	1.29 ± 0.99
TSTRL-cooh → TSTAL-cooh	2.79 ± 0.14	2.42 ± 0.08	0.37 ± 0.16	0.50 ± 1.10
TSTRL-cooh → TRTAL-cooh	2.90 ± 0.19	1.20 ± 0.17	1.70 ± 0.26	0.99 ± 1.27
TSTRL-cooh → ASTRL-cooh	-7.96 ± 0.08	-8.36 ± 0.06	0.40 ± 0.10	0.25 ± 0.88
TSTRL-cooh → TSTTL-cooh (CXCR2)	9.38 ± 0.15	9.38 ± 0.08	-0.01 ± 0.17	0.36 ± 1.27
AQTRL-cooh (SAPAP3) → TQTRL-cooh	0.81 ± 0.09	0.63 ± 0.06	0.19 ± 0.11	-0.01 ± 1.05
AQTRL-cooh (SAPAP3) → TSTRL-cooh	56.86 ± 0.13	55.62 ± 0.07	1.24 ± 0.15	1.03 ± 1.10
AQTRL-cooh (SAPAP3) → TRTAL-cooh	56.62 ± 0.17	55.60 ± 0.12	1.02 ± 0.20	2.02 ± 1.27
AQTRL-cooh (SAPAP3) → ASTRL-cooh	48.81 ± 0.10	47.33 ± 0.07	1.48 ± 0.12	1.28 ± 1.27
ASTTL-cooh → TSTAL-cooh	-2.66 ± 0.10	-2.65 ± 0.07	-0.01 ± 0.12	-0.17 ± 1.10
ASTTL-cooh → ASTRL-cooh	-147.65 ± 0.14	-146.75 ± 0.08	-0.90 ± 0.16	-0.41 ± 1.27
ASTTL-cooh → TSTTL-cooh (CXCR2)	4.24 ± 0.08	4.20 ± 0.08	0.04 ± 0.11	-0.30 ± 1.27
TSTAL-cooh → TSTTL-cooh (CXCR2)	6.67 ± 0.10	6.78 ± 0.10	-0.11 ± 0.13	-0.14 ± 0.53
TRTAV-cooh (MEK2) → AQTRL-cooh (SAPAP3)	-49.57 ± 0.18	-47.18 ± 0.18	-2.38 ± 0.25	-1.99 ± 1.18
TRTAV-cooh (MEK2) → TRTAL-cooh	8.38 ± 0.11	7.70 ± 0.06	0.68 ± 0.12	0.03 ± 1.18

Fig. S4. FEP+ MD simulations of transit between nodes of individual pentamers bound to RGS12 PDZ domain. (**A**) The 9-node/18-edge permutation map networking together the three candidate C-tail ligands (highlighted in yellow, with name of originating protein in parentheses) and six different single amino-acid transformations thereof. Similarity index between two nodes is highlighted on each edge in green. Minimal edges per node (3) and minimal similarities (>0.04) were maintained throughout the network prior to launching FEP+ MD simulations. (**B**) Summary of results obtained for wildtype (WT) and R59Q-variant versions of the RGS12 PDZ domain model (pose 16 of PDB id 2KV8), as compared to GLIDE XP docking scores for the same three C-tail ligands docking into pose 16 of PDB id 2KV8 (Figure 4). (**C, D**) Energy changes (and error estimations) for each node-to-node transit as reported by the FEP+ MD simulations.

Figure S5

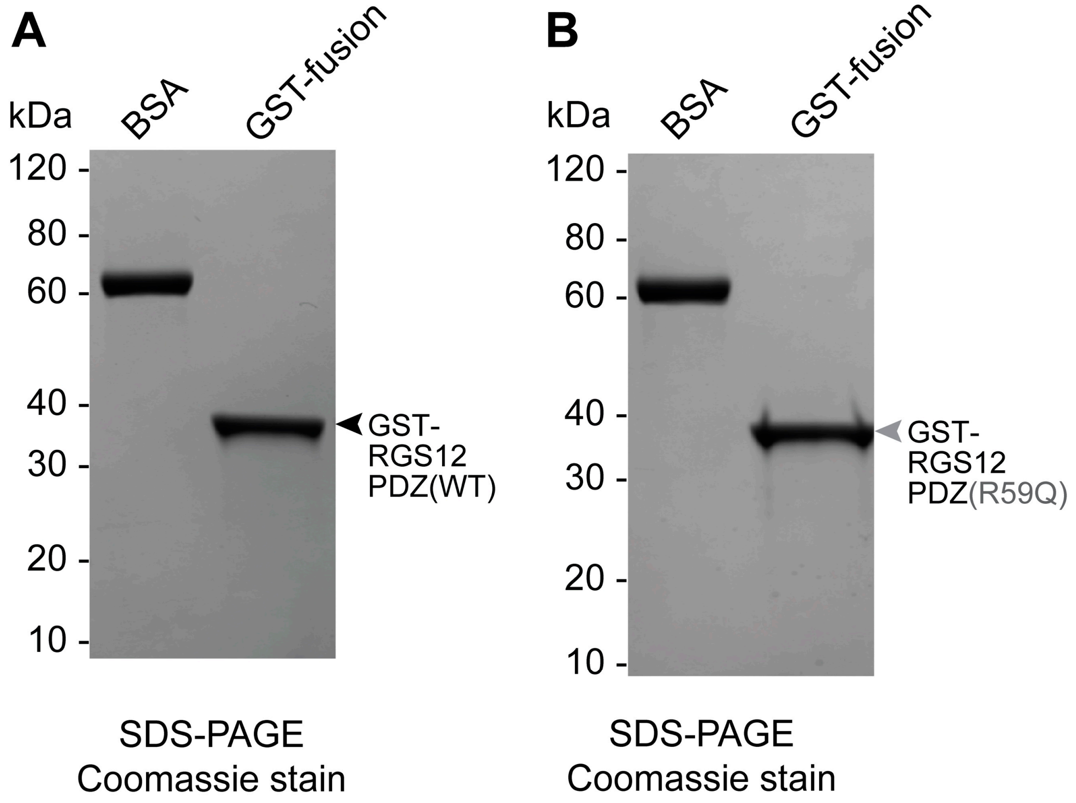

Fig. S5. Regulator of G protein Signaling-12 (RGS12) PDZ domain proteins purified as glutathione-S-transferase (GST) fusions from *Escherichia coli*. Two microgram aliquots of wildtype (panel **A**) or R59Q variant (panel **B**) PDZ domain fusion protein, post purification by high-pressure/high-performance liquid chromatography (HPLC), were separated by sodium dodecyl sulphate (SDS)-polyacrylamide gel electrophoresis, along with molecular weight markers and a control aliquot of two micrograms of bovine serum albumin, and then visualized by Coomassie blue staining.
